# GFAT2-mediated HSPD1 O-GlcNAcylation drives chemotherapy resistance in non-small cell lung cancer

**DOI:** 10.1186/s13046-026-03674-x

**Published:** 2026-02-24

**Authors:** Man Zhu, Xiaoyu Tang, Zeren Zhu, Wenjun Tang, Yumeng Cheng, Wenjuan Tang, Qianqian Zhang, Longyu Qin, Yu Yao, Yanmin Zhang

**Affiliations:** 1 Department of Medical Oncology, the First Affiliated Hospital of Xi’an Jiaotong University, Xi’an, 710061 P. R. China; 2 School of Pharmacy, Health Science Center, Xi’an Jiaotong University, Xi’an, 710061 P.R. China; 3 State Key Laboratory of Shaanxi for Natural Medicines Research and Engineering, Xi’an, 710061 P.R. China

**Keywords:** Non-small cell lung cancer, Chemotherapy resistance, Glutamine-fructose-6-phosphate transaminase 2, O-GlcNAcylation, Heat shock protein family D member

## Abstract

**Background:**

Non-small cell lung cancer (NSCLC) patients often develop resistance to first-line etoposide/cisplatin (EP) chemotherapy. However, available studies only focus on single-agent resistance to either etoposide or cisplatin in NSCLC. Hence, a notable knowledge gap exists in terms of the mechanisms underlying multidrug resistance, particularly within a system that recapitulates EP resistance in NSCLC. This emphasizes an urgent need for new strategies to tackle this challenge.

**Methods:**

This study established a chemotherapy-resistant xenograft mouse model that mimicked the clinical chemotherapy regimens used for patients with NSCLC and aimed to explore the molecular mechanisms that contribute to chemotherapy resistance in NSCLC. The key protein that regulates chemotherapy resistance in NSCLC were identified through proteomics and co-immunoprecipitation mass spectrometry (Co-IP/MS) analyses, and revealed its regulatory mechanisms.

**Results:**

This study identified glutamine-fructose-6-phosphate transaminase 2 (GFAT2,) as a key driver of resistance, upregulated in chemoresistant NSCLC cells. GFAT2 critically regulates the hexosamine biosynthetic pathway (HBP), enhancing uridine diphosphate-GlcNAc (UDP-GlcNAc) synthesis and overall O-GlcNAcylation. Specifically, GFAT2 augments O-GlcNAcylation of heat shock protein family D member 1 (HSPD1) at residue T320. This modification stabilizes HSPD1 by blocking its tripartite motif containing 21 (TRIM21)-mediated ubiquitination and degradation. Stabilized HSPD1 subsequently activates anti-apoptotic signaling, promoting cell survival during chemotherapy. Crucially, knockdown of either GFAT2 or HSPD1 restored chemosensitivity in models.

**Conclusions:**

These findings elucidate the GFAT2/HSPD1 axis and O-GlcNAcylation as pivotal metabolic mechanisms underlying EP resistance, identifying them as promising therapeutic targets to overcome chemoresistance in NSCLC.

**Supplementary Information:**

The online version contains supplementary material available at 10.1186/s13046-026-03674-x.

## Background

Non-small cell lung cancer (NSCLC) is the most common cancer, with a three-year survival rate of only 40% [1]. Although chemotherapy remains the cornerstone of NSCLC treatment, the emergence of chemotherapy resistance markedly diminishes treatment efficacy and leads to poor prognosis. The recommended first-line treatment for locally advanced stage III unresectable NSCLC is concurrent chemoradiotherapy with etoposide and cisplatin (EP) [2]. NSCLC develops heightened resistance to etoposide compared with that of small cell lung cancer [3], with prevalent issues, such as cisplatin resistance, frequently encountered among patients with NSCLC [4, 5]. However, available studies only focus on single-agent resistance to either etoposide or cisplatin in NSCLC. Hence, a notable knowledge gap exists in terms of the mechanisms underlying multidrug resistance, particularly within a system that recapitulates EP resistance in NSCLC. This emphasizes an urgent need for new strategies to tackle this challenge.

Metabolic reprogramming is a recognized cancer marker that promotes tumorigenesis and chemotherapy resistance [6]. The hexosamine biosynthetic pathway (HBP) is a branch of glucose metabolism that plays an important role in tumorigenesis. This pathway is primarily responsible for the synthesis of uridine diphosphate N-acetylglucosamine (UDP-GlcNAc), which is a substrate for O-GlcNAcylation. This process is a post-translational modification that adds N-acetylglucosamine (GlcNAc) to the serine and threonine residues of proteins. O-GlcNAcylation has been implicated in various biological processes, including cell growth, survival, and metabolism, and is particularly relevant in tumor progression [7–9]. For instance, increased glucose metabolism in tumor-associated macrophages fuels O-GlcNAcylation of lysosomal cathepsin B to promote cancer metastasis [10]. Furthermore, O-GlcNAcylation promotes pancreatic cancer progression by regulating protein stability and deacetylation ability [11]. Despite these insights, the exact role of HBP-driven O-GlcNAcylation in NSCLC, especially in chemotherapy resistance, remains unclear.

In this study, proteomics and co-immunoprecipitation mass spectrometry (Co-IP/MS) analyses were used to identify GFAT2 (also denoted as GFPT2) as a key protein that regulates chemotherapy resistance in NSCLC. Moreover, this study demonstrated that GFAT2 mediates the O-glycosylation of HSPD1. Furthermore, GFAT2 appears to be a key rate-limiting enzyme in the HBP, and its catalytic product (UDP-GlcNAc) is an indispensable substrate in the acylation step of O-glycosylation. GFAT2 weakens the phagocytic activity of macrophages by regulating glutamine metabolism, ultimately leading to antibody resistance in patients [12]. However, the role and regulatory mechanism of GFAT2 in tumor chemoresistance have not been clarified. Therefore, Co-IP/MS was used to further explore the mechanism by which GFAT2 regulates chemotherapy resistance. The Co-IP/MS analysis revealed that GFAT2 interacts with HSPD1 and plays a key role in GFAT2-mediated chemotherapy resistance. HSPD1 is a nuclear-encoded mitochondrial chaperone protein that is essential for mitochondrial protein homeostasis. The protein is mainly localized in the mitochondria, and its expression is upregulated in leukemia, breast cancer, colon cancer, lung cancer, and other liquid and solid tumors, and is usually associated with poor prognosis [13]. Nevertheless, the mechanistic crosstalk between HSPD1- and GFAT2-driven hexosamine biosynthesis and their collective impact on chemoresistance remain unclear.

Therefore, this study established a chemotherapy-resistant xenograft mouse model that mimicked the clinical chemotherapy regimens used for patients with NSCLC and aimed to explore the molecular mechanisms that contribute to chemotherapy resistance in NSCLC. Accordingly, this study revealed a previously unknown mechanism in which GFAT2 increased the production of UDP-GlcNAc and O-GlcNAcylation and delineated the functional importance of HSPD1 O-GlcNAcylation in facilitating chemotherapy resistance in NSCLC. The results of this study are expected to offer new therapeutic strategies to combat chemotherapy resistance in NSCLC, ultimately enhancing patient treatment outcomes.

## Methods

### Cell Culture

NCI-H460 (RRID: CVCL_0459), NCI-H1975 (RRID: CVCL_1511), and A549 (RRID: CVCL_0023) cells were cultured in complete medium supplemented with 10% fetal bovine serum, 100 U/mL penicillin, and 100 U/mL streptomycin. NCI-H460 and NCI-H1975 cell lines were cultured in RPMI-1640 medium. A549 cells were cultured in F12K medium. All cell lines were incubated at a constant temperature of 37 °C, with a 5% CO_2_ atmosphere. Subculturing was conducted regularly every 2–3 days to ensure optimal growth conditions. All experiments were conducted using passages 5 to 20 of the cell lines. Prior to use, cells were routinely tested for *Mycoplasma* contamination and confirmed to be negative.

To elucidate the intricate mechanisms underlying chemotherapy resistance in NSCLC, an in vitro chemotherapy-resistant cell line was established using methodical drug concentration-gradient induction. Over a span of five months, NCI-H1975 and NCI-A549 cells were cultured in media supplemented with progressively increasing concentrations of EP. Initially, these cells were subjected to a 48-h incubation with 0.01 µM of etoposide and cisplatin. Thereafter, the cells were transferred to fresh medium without drugs until the cells proliferated to normal morphology. The above steps were repeated 4–6 times for each concentration until the cells could grow stably at that concentration and were then increased to a higher concentration. The RI of the chemotherapy-resistant cells (IC_50_ of induced resistant cells / IC_50_ of parental cells) was analyzed monthly. After five months, human-origin chemotherapy-resistant NSCLC cell lines, designated as H1975 chemotherapy-resistant cells (H1975^EPR^) and A549 chemotherapy-resistant cells (A549^EPR^), were obtained, stably grown, and passaged in 0.16 µM EP.

### Animal models

All animal experiments were performed in accordance with the guidelines of the Institutional Animal Care and Use Committee of the Xi’an Jiaotong University (Project No. XJTUAE2024-1992) and approved by the Ethics Committee of Xi’an Jiaotong University Health Science Center. BALB/c (RRID: MGI:2683685) male nude mice (20 g, four weeks old) were purchased from Jicui Biotechnology and housed under specific pathogen-free (SPF) conditions throughout the experiment.

A cell-derived xenograft (CDX) model of NSCLC was established to generate an EP-resistant xenograft model, and H460 cells were used for model construction. The tumor length and width (mm) were measured using calipers every other day. Briefly, 1 × 10⁶ H460 cells were subcutaneously injected into the armpits of four-week-old nude mice until the tumor volume surpassed 100 mm³. In the EP group, cisplatin (10 mg·kg⁻¹) was administered via intraperitoneal injection on days 1 and 8, whereas etoposide (10 mg·kg⁻¹) was administered from days 1 to 5, with a cycle of four weeks. Normal saline was administered to the control group intraperitoneally. After two consecutive treatment cycles, the tumors were dissected and passaged into naïve nude mice, which continued to undergo EP chemotherapy until they acquired chemoresistance (the tumor volume did not further shrink when given EP treatment). Resected tumor tissues from resistant xenografts were used to isolate and establish EP-resistant cell lines (H460^EPR^) for in vitro culture.

### RNA extraction and RT-qPCR

Total RNA was extracted from cells using a total RNA extraction kit according to the manufacturer’s protocol. Total RNA was reverse-transcribed into complementary DNA using the PrimeScript RT kit (Takara, Kyoto, Japan). The qPCR was performed using an iCycler iQ RT-PCR detection system (Bio-Rad) and SYBR Green qPCR Master Mix (Takara). All reactions were performed in triplicate. The data were analyzed using iCycler software according to the 2−∆Ct method.

### Colony formation assay

Cultured cells were trypsinized to obtain single-cell suspensions and seeded in 24-well plates. When cell clusters consisting of 3–4 cells formed, appropriate treatments were applied. The cells were continuously cultured with fresh complete medium replaced every 2 days until macroscopic colonies were visible. The medium was carefully aspirated, and the cells were rinsed with PBS. Subsequently, the cells were fixed with methanol for 15 min. After discarding the fixative, the cells were rinsed again with PBS, stained with crystal violet solution for 15 min, and then washed twice with PBS and distilled water successively. The plates were air-dried, followed by imaging and counting of the colonies.

### Immunoblotting analysis

Total protein was extracted from samples, and protein concentrations were quantified using a bicinchoninic acid protein assay kit. Protein samples were separated by sodium dodecyl-sulfate polyacrylamide gel electrophoresis and then electrotransferred onto polyvinylidene difluoride membrane. The membrane was blocked with 5% non-fat milk, followed by overnight incubation with primary antibodies at 4℃. After washing with Tris-buffered saline with 0.1% Tween^®^ 20 detergent (TBST), the secondary antibody was incubated at 37℃ for 1 h. Following another The membranes were then washed again with TBST four times (10 min each), incubated with ECL solution and analyzed using a Tanon 5200 automated chemiluminescent imaging analysis system (Tanon, Shanghai, China).

### Immunohistochemistry assay

Tumor tissues were fixed in 4% paraformaldehyde for 12 h. Fixed tissues were embedded in paraffin and cut into 5-µm sections. The sections were dewaxed in xylene, rehydrated in ethanol, rinsed in distilled water, and fixed in 4% formaldehyde. After antigen retrieval in citrate buffer solution (0.01 M, pH 6.0) for 20 min, slides were washed three times with TBS (0.01 M, pH 7.4) and incubated with 1% bovine serum albumin for 1 h. After aspirating blocking serum, tumor tissue sections were incubated with primary antibodies at 4 °C overnight, and then incubated with secondary antibodies for 30 min. The substrate was added to the sections for 30 min, followed by 3,3’-diaminobenzidine staining and Gram staining, and the positive staining was mainly determined according to the brown yellow color in the nucleus. All the sections were imaged using an inverted microscope (Nikon, Tokyo, Japan).

### TUNEL staining assay

Frozen sections with a thickness of 5 μm were processed as previously described. The sections were treated with 4% paraformaldehyde. The cells were then rinsed twice with phosphate-buffered saline (PBS) for 10 min each and treated with 0.1% Triton X-100 in PBS for 2 min on ice. The cells were rinsed with PBS again and incubated with 50 µL of TUNEL reaction solution for 60 min at 37 °C. After washing with PBS, the cells were analyzed under a fluorescence microscope (Olympus, Tokyo, Japan).

### Flow cytometric analysis of apoptosis

Cells were seeded in 6-well plates at a density of 2 × 10^5^ cells/well and cultured overnight at 37 °C with 5% CO₂. On the following day, cells were subjected to designated experimental treatments. After treatment, adherent cells were digested and collected, and combined with floating apoptotic cells harvested from the culture medium. The combined cell suspensions were washed 2–3 times with precooled PBS. Approximately 400 µL of 1 × Annexin V Binding Buffer was added to wash the cells once, the supernatant was then discarded after centrifugation (2000 rpm/min, 5 min). Thereafter, 400 µL of 1 × Annexin V Binding Buffer was added to resuspend the cells, and 5 µL of Annexin V and 10 µL of propidium iodide were added. The cells were then mixed well and placed in ice for 15 min under low-light conditions. Apoptosis rates were quantified immediately using flow cytometry.

### Cell cycle analysis

Cells were seeded in 6-well plates at 5 × 10⁵ cells/well, cultured overnight at 37 °C with 5% CO₂, and treated for 24–48 h. Adherent cells were detached with trypsin-EDTA (without phenol red), combined with floating cells, centrifuged at 300 × g for 5 min at 4 °C, washed twice with precooled PBS, and fixed in 70% ice-cold PBS overnight at 4 °C. Fixed cells were washed twice, resuspended in 500 µL PI/RNase buffer (50 µg/mL PI, 100 µg/mL RNase A), incubated at 37 °C in the dark for 30 min, and analyzed by flow cytometry.

### IP-MS

H460^EPR^ and A549 ^EPR^ cells transfected with OGT-FLAG were collected and incubated with anti-FLAG M2 affinity gel (Sigma-Aldrich, A2220, St. Louis, MI, USA) overnight at 4 °C. The immunoprecipitated complexes were eluted and stained with Coomassie Brilliant Blue. Protein strip samples were cut and sent to Muohe Biotechnology Co., Ltd. (Shanghai, China) for identification of OGT-binding proteins. After reduction and alkylation, the samples were incubated with trypsin (mass ratio 1:50). After desalination and lyophilization, trypsin products were dissolved in 0.1% formic acid solution. MS was performed using an O Exactive HF MS instrument coupled with a Dionex ultimate3000 LC instrument. Raw MS data were analyzed using mascot 2.2 software to identify immunoprecipitated proteins. Pathway enrichment analysis was performed using the Kyoto Encyclopedia of Genes and Genomes (KEGG) database (http://kobas.cbi.pku.edu.cn/).

### Proteomics

Established chemoresistant NSCLC xenograft tumors were mechanically minced and digested into single-cell suspensions, which were cultured in vitro for 3–5 passages. Cells were harvested when they reached a stable state, centrifuged to remove the supernatant, snap frozen in liquid nitrogen, and stored at − 80℃. Four volumes of lysis buffer (8 M urea and 1% protease inhibitor) were added to the samples, which were lysed by sonication. After centrifugation at 12,000 g for 10 min at 4℃, cell debris were removed, and the supernatant was enzymatically hydrolyzed. Trichloroacetic acid was added, the mixture was centrifuged, and the supernatant was discarded. After drying, tetraethylammonium bromide at a final concentration of 200 mM was added, and the precipitate was dispersed via ultrasound. Trypsin was added at a ratio of 1:50 (protease: protein, m/m) and the samples were enzymatically hydrolyzed overnight. Dithiothreitol was added to a final concentration of 5 mm and reduced at 56℃ for 30 min. Iodoacetamide was added at a final concentration of 11 mM and incubated at room temperature in the dark for 15 min. Finally, the samples were desalted using a Strata XSPE column. The samples were dissolved in solvent A (0.1% formic acid and 2% acetonitrile/water) and proteomic analysis was performed using ultra-performance liquid chromatography (Bruker Daltonics) combined with time-of-flight mass spectrometry (timsTOF Pro).

### Metabolomics

The parental cells and chemoresistant cells were seeded into 100-mm dishes at a density of 6 × 10^5^ cells per dish, washed twice with precooled PBS after 24 h of culture, collected into centrifuge tubes, snap frozen in liquid nitrogen, and stored at − 80 °C. Samples were sent to Shanghai Baipu Biotechnology Co., Ltd. on dry ice and untargeted metabolomic analysis was performed using a UPLC-ESI-Q-Orbitrap-MS system (UHPLC, Shimadzu Nexera X2 LC-30AD, Shimadzu, Japan) combined with Q-Exactive Plus (Thermo Scientific, San Jose, USA). For liquid chromatography separation, the samples were subjected to analysis via an ACQUITY UPLC^®^ HSS T3 column (2.1 × 100 mm, 1.8 μm) (Waters, Milford, MA, USA). Positive- and negative-mode electrospray ionization were then used for mass spectrometry data acquisition.

### Statistical analysis

All statistical analyses were performed using GraphPad Prism 8 software (GraphPad Software Inc.). Experimental data are presented as mean ± standard error mean. At least three independent experiments were performed. Student’s t-test was used for comparison between two groups, Tukey’s test was used for comparison between multiple groups after one-way ANOVA, and Pearson’s test was used for correlation analysis, with *p* < 0.05 considered statistically significant.

## Results

### Construction and analysis of EP-resistant NSCLC model

**Fig. 1:**
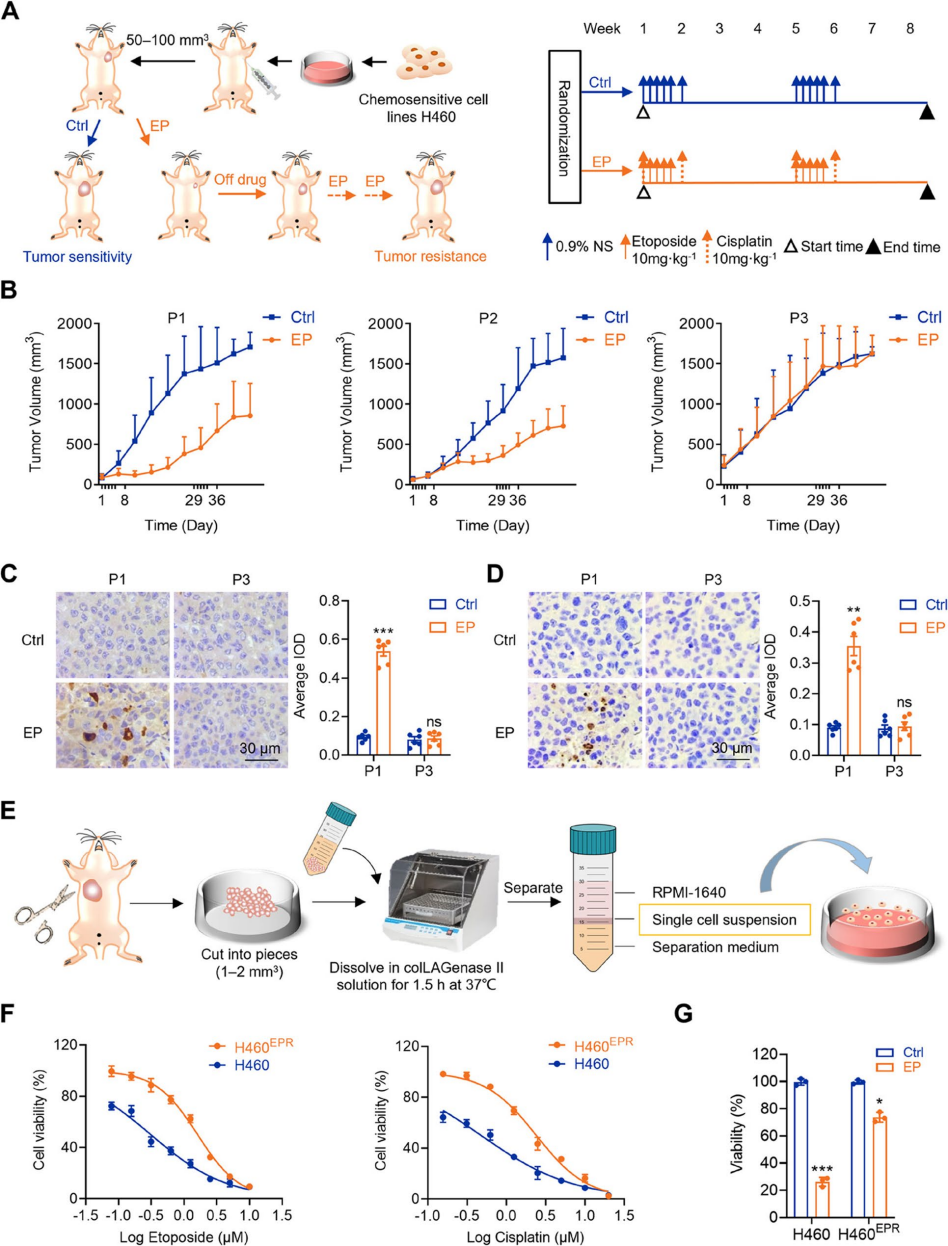
Establishment and validation of in vivo chemoresistant cell line-derived xenograft mouse models. **A** Schematic of the establishment of chemoresistant CDX mouse models using human NSCLC cell lines. **B** Tumor growth kinetics of NSCLC chemosensitive xenograft models (H460) in response to etoposide and cisplatin (EP) treatment in different passages. Different passages of tumor serial xenografts are separated by dashed lines. Ticks on the x-axis indicate day 1 of the weekly EP cycle. Data are shown as average tumor volumes of the control (Ctrl) group and EP group. Px represents Passage x: P1, *n* = 6; P2, *n* = 6; P3, *n* = 6. **C**–**D** Immunohistochemistry staining and quantification of (**C**) cleaved caspase-3 and (**D**) γH2AX. **E** Schematic diagram showing establishment of in vivo chemoresistant NSCLC cell line. **F** Relative cell viability of chemosensitive (H460) and chemoresistant (H460^EPR^) cells upon treatment with different etoposide and cisplatin concentrations for 48 h. **G** Relative viability of H460 and H460^EPR^ cells following 48 h of EP treatment (0.16 µM etoposide and 0.24 µM cisplatin) in vitro. Data are shown as mean ± SEM (*n* = 3 biological replicates for cell experiments; *n* = 6 biological replicates for animal experiments). *p < 0.05, **p < 0.01, ***p < 0.001, ns, no significance

To investigate chemotherapy resistance in NSCLC in a living organism, a cell line-derived xenograft (CDX) model was established using three cycles of EP treatment until the tumors developed chemotherapy resistance (Fig. 1A, B). The levels of cleaved caspase-3 and γH2AX markedly decreased in tumors from the chemotherapy group at P3 (Fig. 1C, D). This further supported the conclusion that in vivo chemotherapy resistance was acquired.

To evaluate whether cells derived from a chemotherapy-resistant model in vivo also displayed resistance in vitro, chemotherapy-resistant NSCLC xenograft tumors were isolated and digested. Subsequently, these cells were cultured and designated H460^EPR^ cells (Fig. 1E). Cell viability assays indicated that H460^EPR^ cells showed markedly higher viability than that of H460 cells following etoposide treatment, with half-maximal inhibitory concentration (IC_50_) values of 1.630 µM for H460^EPR^ and 0.3117 µM for H460 cells. A similar trend was observed with cisplatin, where the IC_50_ values for H460^EPR^ and H460 cells were 2.412 µM and 0.4747 µM, respectively. The resistance index for both drugs exceeded 5 in H460^EPR^ cells (Fig. 1F), thus signifying a substantial level of drug resistance to both etoposide and cisplatin. Given all subsequent mechanistic experiments in this study adopted the combined EP treatment, we further validated EP resistance at a rationally selected concentration (etoposide: 0.16 µM; cisplatin: 0.24 µM) based on the above single-agent IC_50_ data. Furthermore, as illustrated in Fig. 1G, H460^EPR^ cells demonstrated markedly reduced sensitivity to EP compared with that of H460 cells. These findings confirmed the successful establishment of a chemotherapy-resistant NSCLC cell line in vivo.

### GFAT2 is a critical mediator of chemotherapy resistance in NSCLC

**Fig. 2:**
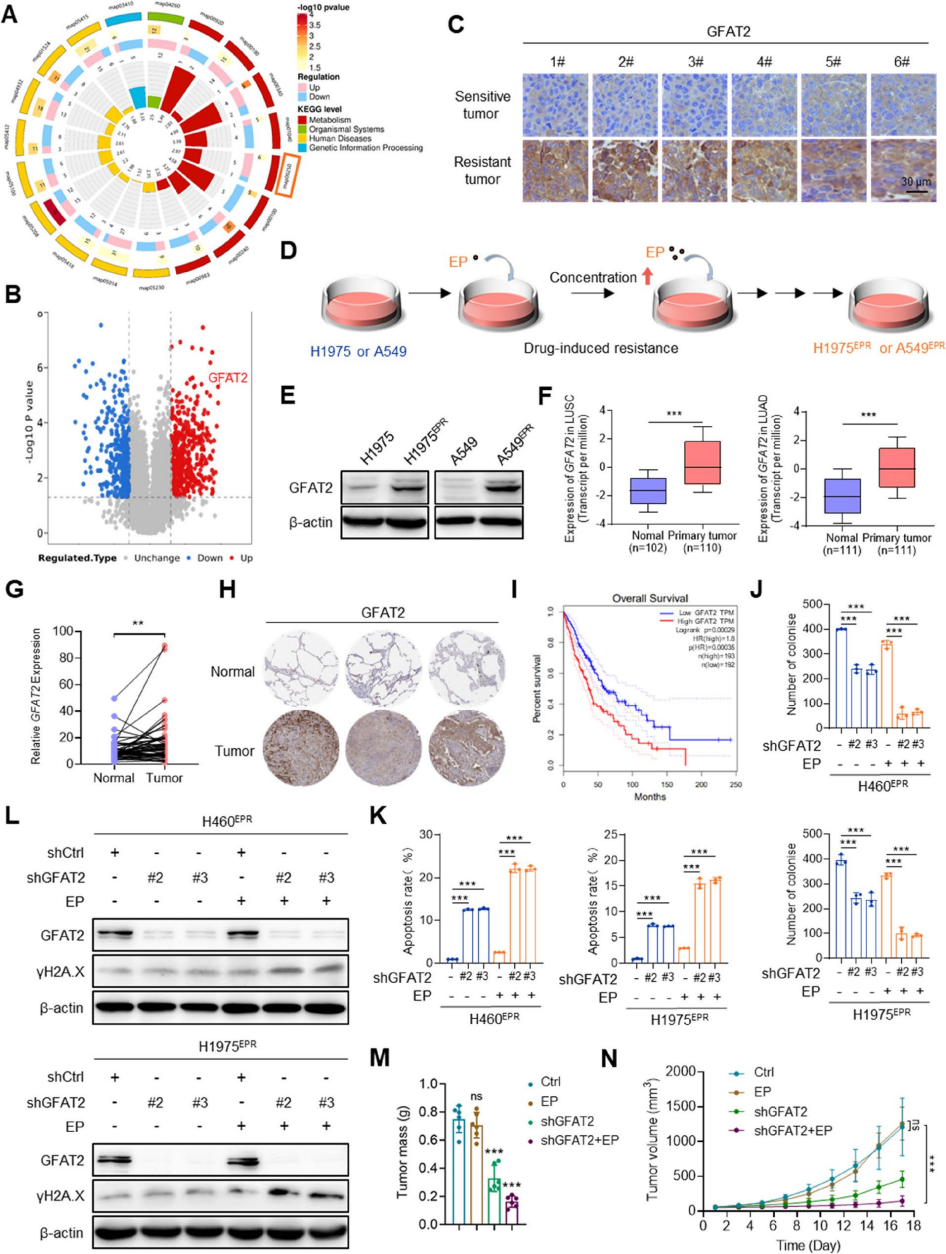
GFAT2 is a critical mediator of chemotherapy resistance in NSCLC.**A** KEGG pathway Circos diagram of differential proteins between chemoresistant cells and chemosensitive cells. **B** Volcano diagram of differential proteins between chemoresistant cells and chemosensitive cells. Red dots represent upregulated proteins, blue represents downregulation, and gray represents no significant difference. Fold-change (*FC*) > 1.5, *P* value < 0.05. **C** Immunohistochemistry staining of GFAT2 in chemotherapy-sensitive and -resistant tumors derived from cell line-derived xenograft (CDX) models (×400). **D** Schematic diagram showing the establishment of chemoresistant NSCLC cell lines in vitro. **E** Immunoblotting analysis of GFAT2 in chemoresistant NSCLC cell lines and parental cell lines. **F**–**G** Expression of *GFAT2* in NSCLC tissues from The Cancer Genome Atlas Program (TCGA) database. **F** lung adenocarcinoma (LUAD, left) and lung squamous cell carcinoma (LUSC, right); (**G**) 110 samples of normal and tumor tissues from the same patient. **H** Expression of GFAT2 in normal and tumor tissues in Human Protein Atlas (HPA) database (×4). **I** Kaplan-Meier survival curve representing overall survival in chemotherapy-treated basal patients with NSCLC based on different GFAT2 expression levels from Gene Expression Profiling Interactive Analysis (GEPIA) database. **J** Colony formation capacity of H460^EPR^ and H1975^EPR^ cells with *GFAT2* knockdown (shGFAT2), with or without etoposide and cisplatin (EP) treatment. **K** Apoptosis rate of H460^EPR^ and H1975^EPR^ cells with *GFAT2*-knockdown in the presence or absence of EP. **L** Immunoblotting analysis of γH2A.X in H460^EPR^ and H1975^EPR^ cells with *GFAT2* knockdown, with or without EP treatment. **M**–**N** Effect of *GFAT2*-knockdown on (**M**) tumor mass and (**N**) volume in H460^EPR^ cells with or without EP treatment. Mice were treated with vehicle control or EP (10 mg/kg). Data shown as mean ± SEM (*n* = 3 biological replicates for cell experiments; *n* = 6 biological replicates for animal experiments). **p < 0.01, ***p < 0.001, ns, no significance

Proteomic profiling identified 564 upregulated and 347 downregulated proteins in chemotherapy-resistant cells versus -sensitive cells (Fig. S1A). Kyoto Encyclopedia of Genes and Genomes (KEGG) pathway enrichment analysis revealed significant enrichment in metabolic pathways, particularly alanine, aspartate, and glutamate metabolism (KEGG: 00250) (Fig. 2A, S1B). By focusing on the top 5 most highly upregulated proteins and integrating univariate and multivariate Cox regression analyses, we identified GFAT2—the rate-limiting enzyme of the hexosamine biosynthetic pathway (HBP)—as a core target, as it was the only one validated as an independent prognostic risk factor for poor survival in NSCLC patients (Fig. S1C, D). A volcano plot further also highlighted GFAT2 as one of the most substantially upregulated proteins in resistant cells (Fig. 2B). Immunohistochemical staining confirmed elevated GFAT2 expression in chemotherapy-resistant tumor samples (Fig. 2C), and GFAT2 was also markedly upregulated in chemotherapy-resistant cells derived from CDX models compared with that in their sensitive counterparts (Fig. S1E).

Additionally, GFAT2 protein expression was progressively increased in NSCLC cell lines (H460, H1975, A549) upon prolonged EP exposure (Fig. S1F). Next, chemotherapy-resistant cell lines (H1975^EPR^ and A549^EPR^) were established via continuous low-dose drug induction and monitoring of drug resistance phenotypes (Fig. 2D). Cell viability assays confirmed that both lines exhibited a RI > 5 to etoposide and cisplatin (Fig. S1G, H), and were significantly more resistant to EP than their parental counterparts (Fig. S1I). This confirmed the successful establishment of chemotherapy-resistant NSCLC cell lines in vitro. Western blotting revealed marked upregulation of the multidrug resistance-associated transporters (P-glycoprotein and MRP1) in H460^EPR^ and A549^EPR^ cells (Fig. S2A). Cell cycle distribution assays further showed that EP induced a prominent G2/M phase arrest in parental cells (a hallmark of EP-mediated DNA damage and apoptosis), whereas resistant cells evaded this checkpoint blockade (Fig. S2B), consistent with the core features of chemoresistant cells. Notably, GFAT2 protein levels were also significantly elevated in H1975^EPR^ and A549^EPR^ cells relative to their parental lines (Fig. 2E).

In addition, we investigated the expression and prognostic implications of GFAT2 in NSCLC patients via database analysis. The Cancer Genome Atlas Program database confirmed that *GFAT2* was significantly overexpressed in clinical NSCLC tissues (Fig. 2F). Paired samples analysis further revealed that *GFAT2* expression was notably elevated in tumor tissues compared with adjacent normal lung tissues from the same patients (Fig. 2G). The Human Protein Atlas database corroborated this finding, showing marked overexpression of GFAT2 protein in NSCLC tumors relative to normal lung tissues (Fig. 2H). Survival analysis indicated that NSCLC patients with high GFAT2 expression had considerably shorter overall survival than those with lower expression levels (Fig. 2I). Both univariate and multivariate analyses established that the upregulation of GFAT2 in tumor tissues serves as an independent risk factor for NSCLC (Fig. S1J).

To understand the molecular mechanisms underlying chemoresistance in NSCLC, we established *GFAT2*-knockdown models (Fig. S3A, B). GFAT2 depletion led to a pronounced reduction in the ability of resilient cells to form colonies, particularly in the absence of EP treatment. Notably, combined *GFAT2* knockdown and EP treatment further enhanced the anti-proliferative effect, exceeding the efficacy of EP alone (Fig. 2J, S3C, D). Additionally, *GFAT2* knockdown markedly increased the apoptotic response of EP-treated chemoresistant cells (Fig. 2K, S3E, F). Western blotting revealed that EP treatment did not cause notable DNA damage in chemoresistant cells. However, GFAT2 depletion greatly amplified the DNA damage induced by EP (Fig. 2L). Collectively, these findings suggest that GFAT2 knockdown may restore the sensitivity of chemoresistant cells to EP.

To further elucidate the role of GFAT2 in chemosensitivity, a mouse xenograft model of H460^EPR^ cells with/without GFAT2 depletion was established. GFAT2 depletion led to a decrease in tumor size, weight, and volume in H460^EPR^ cells (Fig. 2M, N and Fig. S3G, H). Notably, the combination of GFAT2 depletion and EP treatment produced a substantially stronger inhibitory effect on tumor growth than either treatment alone,. demonstrating that GFAT2 contributes to chemoresistance in NSCLC tumors and its depletion reverse resistance to EP (Fig. 2M, N and Fig. S3G, H).

### GFAT2 regulates O-GlcNAc Transferase (OGT) and O-linked N-acetylglucosaminase (OGA) to enhance O-GlcNAcylation levels in chemotherapy-resistant NSCLC cells

GFAT2 is an important rate-limiting enzyme in the HBP that mediates the O-GlcNAcylation of intracellular proteins. The aberrant expression of GFAT2 has been observed in chemotherapy-resistant NSCLC cells. To elucidate the ramifications of O-GlcNAcylation on chemoresistance, untargeted metabolomic profiling was employed to analyze the metabolic landscape of chemotherapy-resistant NSCLC cells.

**Fig. 3:**
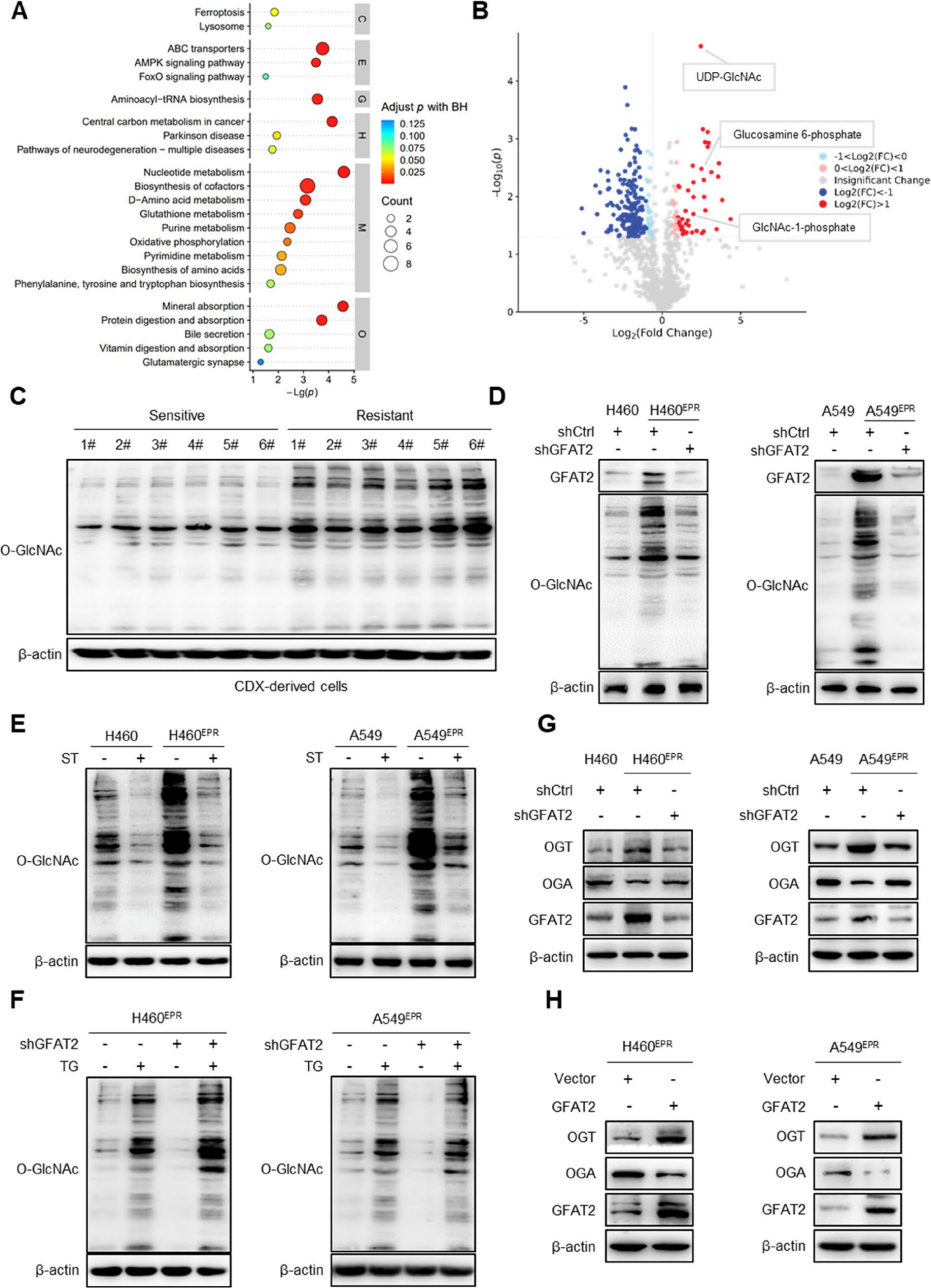
GFAT2 regulates OGT and OGA to enhance O-GlcNAcylation levels in chemotherapy-resistant NSCLC cells. **A** KEGG pathway bubble plot of differential metabolites between chemoresistant and chemosensitive cells. **B** Volcano plot of fold-change including HBP metabolites. **C** Immunoblotting analysis of O-GlcNAcylation in chemotherapy-sensitive and -resistant tumors. **D** Immunoblotting analysis of protein O-GlcNAcylation levels with *GFAT2* knockdown in chemoresistant cells. **E** Immunoblotting analysis of protein O-GlcNAcylation levels with ST045849 treatment (ST, OGT inhibitor, 50 µM) in chemoresistant and parental cells. **F** Immunoblotting analysis of protein O-GlcNAcylation levels with thiamet G treatment (TG, OGA inhibitor, 25 µM) and *GFAT2* knockdown in chemoresistant cells. **G** Immunoblotting analysis of OGT and OGA with *GFAT2* knockdown in chemoresistant and parental cells. **H** Immunoblotting analysis of OGT and OGA with GFAT2 overexpression in chemoresistant cells

Untargeted metabolic profiling of chemotherapy-resistant NSCLC cells revealed upregulation of HBP-related pathways, including purine and pyrimidine synthesis, accompanied by elevated levels of O-GlcNAcylation intermediates (GlcN-6-P, GlcNAc-1-P, UDP-GlcNAc) (Fig. 3A, B, S4A). Western blotting confirmed marked upregulation of O-GlcNAcylation levels in chemotherapy-resistant cells relative to their sensitive counterparts (Fig. 3C). Similarly, protein O-GlcNAcylation levels were markedly increased in H460^EPR^ and A549^EPR^ cells, with *GFAT2* knockdown leading to reduced O-GlcNAcylation levels (Fig. 3D).

OGT and OGA act as the sole regulators of O-GlcNAc addition and removal, respectively (Fig. S4B). We next determined whether GFAT2-mediated O-GlcNAcylation in NSCLC cells was dependent on OGT and OGA. OGT inhibition with ST045849 (ST) reduced O-GlcNAcylation in both parental and chemotherapy-resistant cells, indicating that GFAT2-regulated O-GlcNAcylation is OGT-dependent (Fig. 3E). Furthermore, application of the OGA inhibitor thiamet G (TG) to H460^EPR^ and A549^EPR^ cells resulted in an increase in O-GlcNAcylation levels, whereas the inhibition of OGA reversed the suppressive effect of *GFAT2* knockdown on cellular O-GlcNAcylation (Fig. 3F). GFAT2-mediated O-GlcNAcylation in chemotherapy-resistant cells is similarly regulated by OGA. These experimental findings indicate that GFAT2 modulates cellular O-GlcNAcylation by regulating OGT and OGA proteins.

Moreover, the study investigated whether GFAT2 influences O-GlcNAcylation levels in H460^EPR^ and A549^EPR^ cells by modulating the expression of OGT and OGA. Relative to parental cells, resistant cells showed elevated OGT expression, coupled with a reduction in OGA expression. Furthermore, *GFAT2* knockdown markedly decreased OGT and increased OGA expression, whereas GFAT2 overexpression produced the opposite effects (Fig. 3G, H). Overall, GFAT2 primarily orchestrates the regulation of intracellular protein O-GlcNAcylation through its influence on OGT and OGA protein levels.

### OGT and OGA mediate the chemoresistance effect of GFAT2 in NSCLC cells

**Fig. 4:**
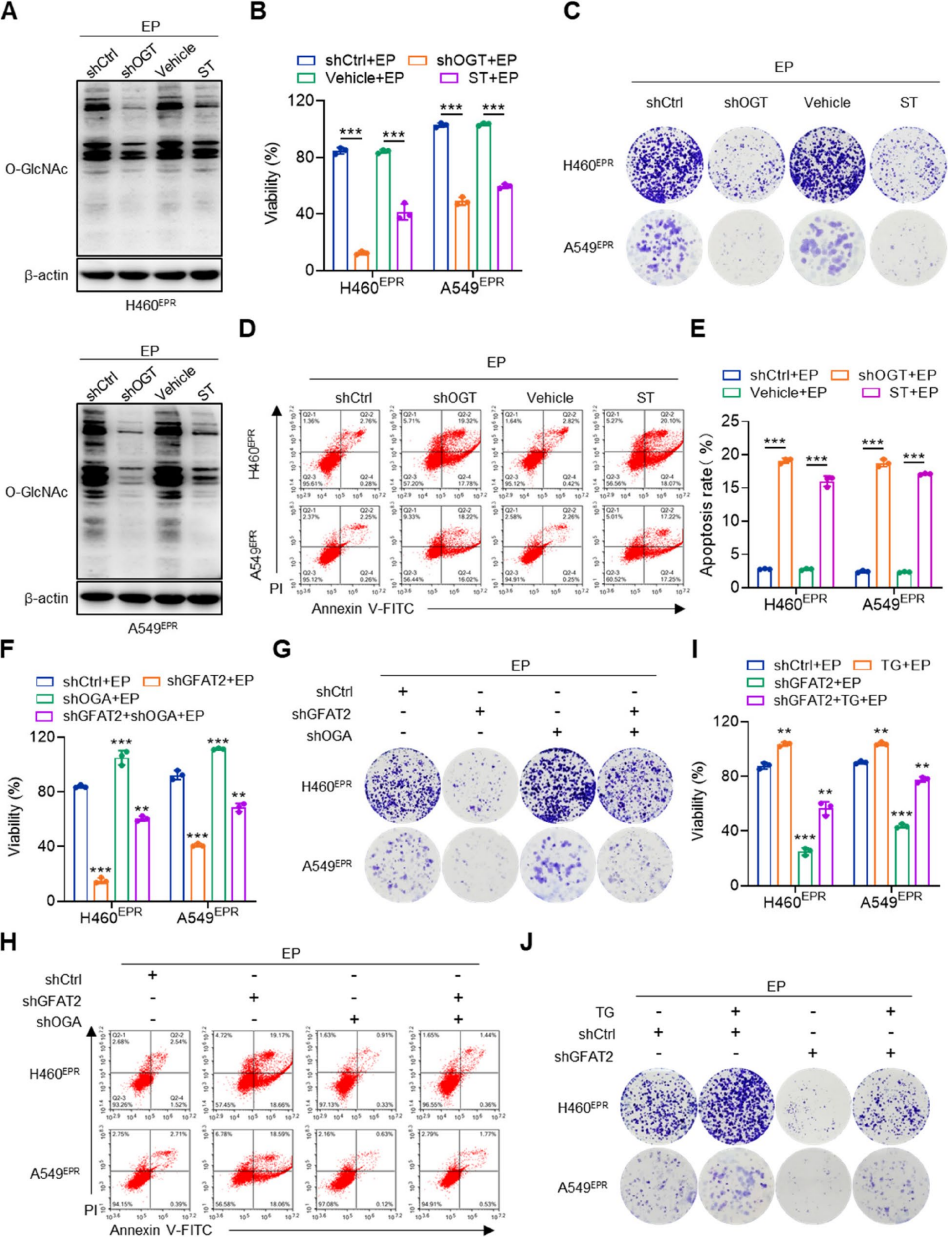
OGT and OGA mediate the chemoresistance effect of GFAT2 in NSCLC cells. **A** Immunoblotting analysis, (**B**) relative viability, (**C**) colony formation capacity, and (**D**–**E**) apoptosis rate of H460^EPR^ and A549^EPR^ cells with ST045849 (ST) or *OGT* knockdown in the presence of etoposide and cisplatin (EP). **F** Relative viability, (**G**) colony formation capacity and (**H**) apoptosis rate of H460^EPR^ and A549^EPR^ cells with *OGA* and *GFAT2* knockdown in the presence of EP. **I** Relative viability and (**J**) colony formation capacity of H460^EPR^ and A549^EPR^ cells with thiamet G (TG) or *GFAT2* knockdown in the presence of EP. Data shown as mean ± SEM (*n* = 3 biological replicates). **p < 0.01, ***p < 0.001

The intricate interplay between OGT and OGA was comprehensively investigated by exploring their influence on the chemoresistance of NSCLC cells. Western blotting revealed a compelling narrative: under the dual influence of EP, both the silencing of OGT and the application of ST effectively curtailed the O-GlcNAcylation levels in drug-resistant cells (Fig. 4A). Concurrently, *OGT* knockdown or inhibition via ST amplified the effect of EP, enhancing the suppression of cell viability, clone formation, and induction apoptosis in resistant cells.(Fig. 4B–E). Although silencing GFAT2 restored the sensitivity of drug-resistant cells to EP, *OGA* knockdown abrogated the effects of EP on cell viability, suppression of clone formation, and induction of apoptosis within these tenacious cells (Fig. 4F–H, S5A). Similarly, *GFAT2* knockdown not only restored the effects of EP on cell viability but also inhibited clone formation and the induction of apoptosis. Notably, application of TG similarly reversed these effects (Fig. 4I-J, S5B-C). These findings elucidate the pivotal roles of both OGT and OGA in the chemoresistance orchestrated by the aberrant expression of GFAT2 in NSCLC.

### OGT mediates O-GlcNAcylation at the T320 site of HSPD1

**Fig. 5:**
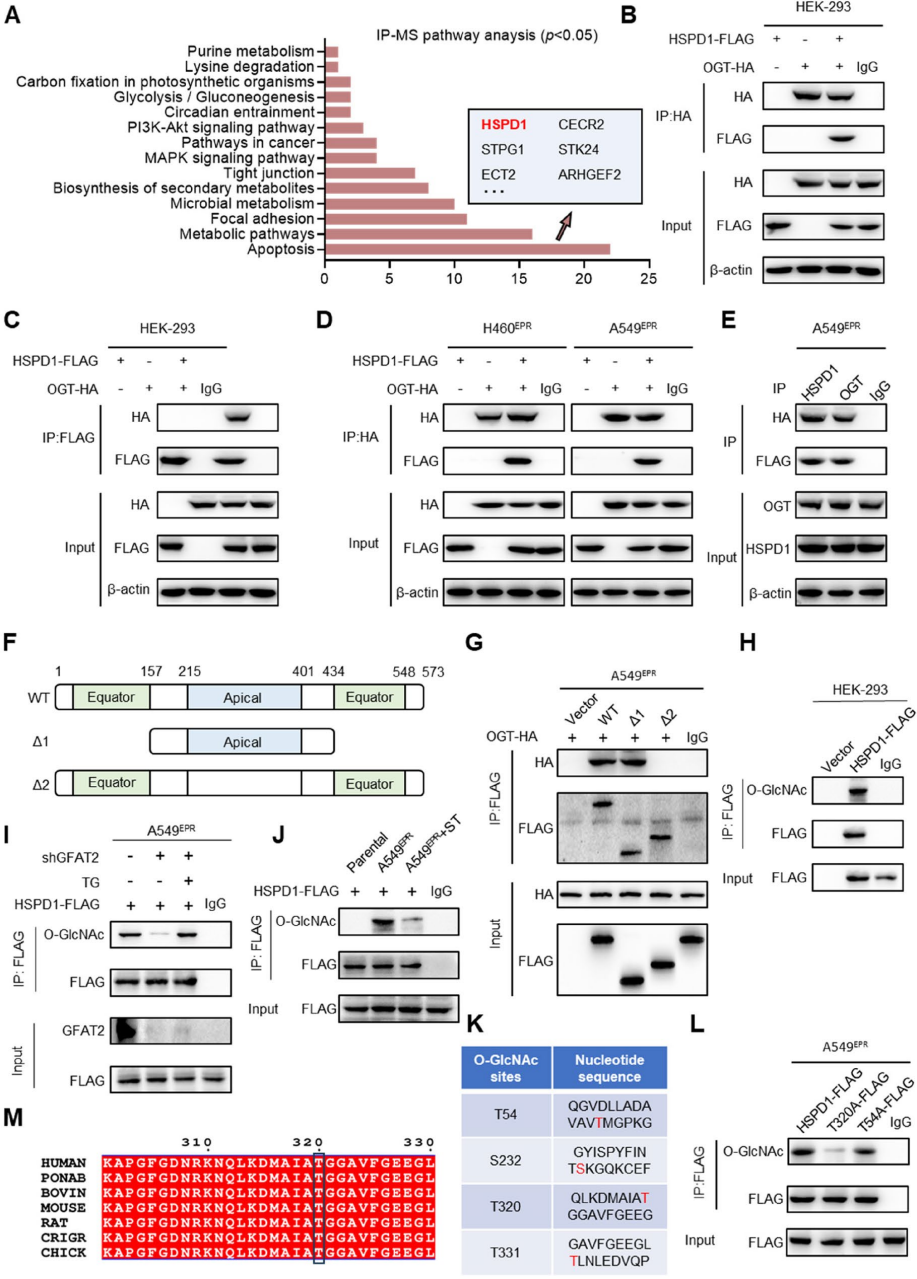
OGT mediates O-GlcNAcylation at the T320 site of HSPD1. **A** KEGG pathway bar plot of proteins that bound to FLAG-tagged OGT in H460^EPR^ and A549^EPR^ cells after immunoprecipitation coupled with mass spectrometry (IP-MS) analysis. **B**–**C** Co-immunoprecipitation (Co-IP) of OGT-HA and HSPD1-FLAG was performed with (**B**) anti-HA antibody or (**C**) anti-FLAG antibody in HEK-293 cells. **D** Co-IP of OGT-HA and HSPD1-FLAG was performed with anti-HA antibody in H460^EPR^ and A549^EPR^ cells. **E** Co-IP of endogenous OGT and HSPD1 in A549^EPR^ cells. **F** Schematic representation of the HSPD1 constructs. Wild-type (WT) HSPD1 contains three domains, including two equator domains, an apical domain, and two median domains. Truncation mutants of HSPD1, comprising 158–433 or 1-214-402-573, were designated as Δ1 and Δ2, respectively. **G** Interactions between OGT and full-length WT, the Δ1 or the Δ2 in A549^EPR^ cells were determined via Co-IP. **H** Co-IP of HSPD1-FLAG was performed with anti-O-GlcNAc antibody or anti-FLAG antibody in HEK-293 cells. **I**–**J** Co-IP of HSPD1-FLAG was performed with anti-O-GlcNAc antibody or anti-FLAG antibody in A549^EPR^ cells and parental cells. **K** Predicted O-GlcNAc sites of HSPD1. **L** Co-IP of HSPD1-FLAG, T320A-FLAG, and T54A-FLAG was performed with anti-O-GlcNAc antibody or anti-FLAG antibody in A549^EPR^ cells. **M** Cross-species sequence alignment of HSPD1

To further elucidate the mechanism by which OGT-mediated protein O-GlcNAcylation promotes EP resistance in H460^EPR^ cells overexpressing GFAT2, immunoprecipitation coupled with mass spectrometry (IP-MS) was performed screen for proteins that exhibit specific interactions with OGT in H460^EPR^ cells. In total, 135 candidate proteins that bind to OGT were identified. Subsequent pathway enrichment analysis of the IP-MS data revealed the involvement of these proteins in critical processes, such as apoptosis, metabolism, and focal adhesion (Fig. 5A).

Focusing on the apoptosis-related protein subset, 22 OGT-interacting proteins were identified, including PPP2R1A, HSPD1, IFIT2, SLC25A5, LMNA, EI24, ARHGEF2, STPG1, STK24, SENP1, ECT2, CECR2, TCHP, UMODL1, PLK1, HSPA9, RASA1, YBX3, NLRP2B, KRT8, GAPDH and NOS1AP. Comprehensive bioinformatics analyses of these 22 proteins in lung adenocarcinoma and lung squamous cell carcinoma with three stringent criteria: gene and protein expression patterns in tumor tissues versus adjacent normal tissues, protein expression across different pathological grades, and correlations between gene expression and patient overall survival. Results indicated that only HSPD1 met all criteria: its gene and protein levels were significantly upregulated in NSCLC, its protein expression was positively correlated with increasing pathological grades of lung adenocarcinoma, and high HSPD1 expression was associated with shorter overall survival in lung adenocarcinoma patients (Fig. S6).

HSPD1, a nuclear-encoded mitochondrial chaperone critical for mitochondrial protein homeostasis, was identified as the core target for subsequent mechanistic investigation. Co-IP experiments confirmed the interaction between HSPD1 and OGT in HEK293 cells co-expressing FlAG-tagged HSPD1 and hemagglutinin (HA)-tagged OGT (Fig. 5B, C). This interactions was further validated in H460^EPR^ and A549^EPR^ cells overexpressing these tagged proteins (Fig. 5D) and in endogenously expressed proteins in A549^EPR^ cells (Fig. 5E). To determine the binding domain of HSPD1, HSPD1 truncation mutants Δ1 (158–433aa) and Δ2 (1–214aa + 402–573aa) were constructed. Co-IP assays revealed that OGT binds to wild-type HSPD1 and Δ1, but not to the Δ2 (Fig. 5F-G). Given that Δ1 encompasses the middle and apical domains, whereas Δ2 comprises the equatorial and middle domains, and considering the binding affinity of OGT for Δ1, OGT specifically binds to the apical domain of HSPD1.

Next, we determined whether HSPD1 undergoes O-GlcNAcylation. Immunoprecipitation revealed that HSPD1 in HSPD1-FLAG-overexpressing HEK293 cells is indeed O-GlcNAcylation (Fig. 5H). Further investigation revealed that *GFAT2* knockdown in H460^EPR^ and A549^EPR^ cells reduces HSPD1 O-GlcNAcylation levels, while OGA inhibition increases these levels (Fig. 5I). In parental and drug-resistant cells overexpressing HSPD1-FLAG and treated with ST, immunoprecipitation experiments confirmed elevated HSPD1 O-GlcNAcylation in drug-resistant cells, with OGT inhibition correspondingly reducing these levels (Fig. 5J).

The precise locus of HSPD1 O-GlcNAcylation was delineated. The O-GlcNAcylation Database indicated four potential sites: T54, S232, T320, and T331 (Fig. 5K), with S232 and T331 lacking cross-species conservation. We constructed HSPD1 point mutation plasmids (T54A-FlAG and T320A-FlAG). Mutational analysis revealed a notable reduction in O-GlcNAcylation in the T320A mutant, whereas T54A mutant remained largely unchanged (Fig. 5L), confirming T320 as the major O-GlcNAcylation site of HSPD1. Comparative analysis of the amino acid sequences of HSPD1 across various species revealed that the T320 site and its adjacent amino acids were markedly conserved among vertebrates (Fig. 5M). In summary, HSPD1 engages with OGT and undergoes O-GlcNAcylation. In chemoresistant NSCLC, the aberrant expression of GFAT2 culminates in an elevation of HSPD1 O-GlcNAcylation.

### The T320-O-GlcNAcylation of HSPD1 affects the TRIM21-mediated degradation of HSPD1

**Fig. 6:**
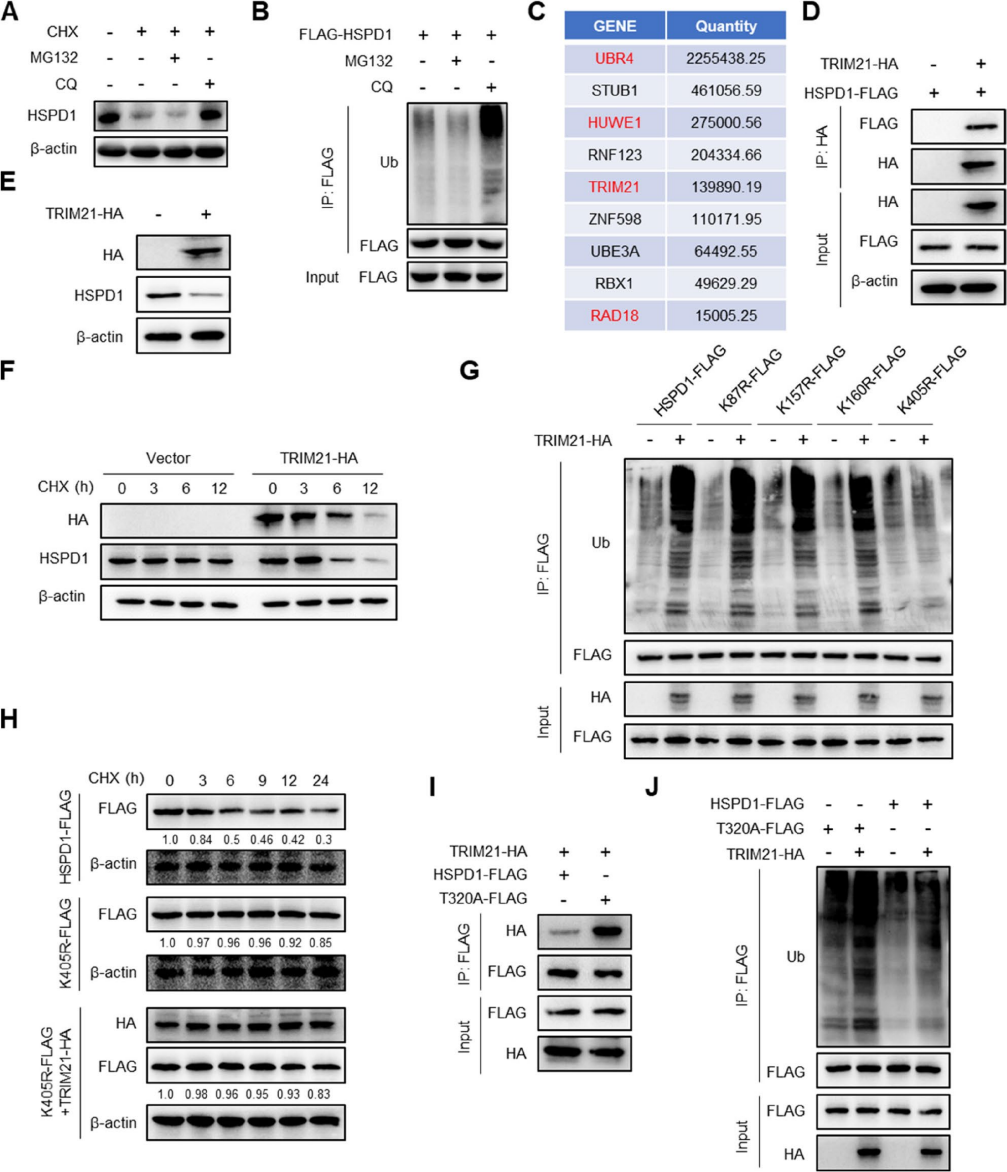
The T320-O-GlcNAcylation of HSPD1 affects the TRIM21-mediated degradation of HSPD1. **A** Cycloheximide (CHX, 20 µg mL^− 1^) was added to A549^EPR^ cells for 24 h, and chloroquine (CQ, 20 μm) or MG132 (20 μm) was added simultaneously. HSPD1 expression was detected using western blotting. **B** FLAG-HSPD1 was overexpressed in A549^EPR^ cells treated with MG132 (20 μm) or CQ (20 μm) for 24 h. Co-IP and western blotting were used to detect the ubiquitination level of HSPD1. **C** The E3 ligase for HSPD1 identified by mass spectrometry is shown. **D** The interaction between HSPD1 and TRIM21 was detected usingCo-IP in A549^EPR^ cells transfected with FLAG-HSPD1 and HA-TRIM21. **E** A549^EPR^ cells were transfected with hemagglutinin (HA)-TRIM21, and western blotting was used to detect the expression of the indicated proteins. **F** A549^EPR^ cells transfected with or without HA-TRIM21 were treated with CHX (20 µg mL^− 1^) for the indicated times. The protein stability of HSPD1 was detected via western blotting. **G** A549^EPR^ cells were transfected with the indicated plasmids, and the ubiquitination of HSPD1 was detected via Co-IP and western blotting. **H** A549^EPR^ cells were transfected with the indicated plasmids, then treated with CHX (20 µg mL^− 1^) for 0, 3, 6, 9, 12, and 24 h. The degradation rate of HSPD1 was detected via western blotting. **I** A549^EPR^ cells were transfected with the indicated plasmids, and Co-IP and western blotting were used to detect the interaction between HSPD1 and TRIM21. **J** A549^EPR^ cells were transfected with the indicated plasmids. The ubiquitination of the HSPD1 protein was detected via Co-IP and western blotting

To investigate the degradation manner of HSPD1, A549^EPR^ cells were subjected to cycloheximide (CHX) for 24 h, which resulted in a pronounced decline in HSPD1 protein levels. This decline was reversible upon addition of the lysosomal inhibitor chloroquine (CQ), whereas the proteasome inhibitor MG132 exhibited no restorative influence (Fig. 6A). CQ markedly augmented HSPD1 ubiquitination, whereas MG132 had no effect (Fig. 6B). Mass spectrometry (MS) analysis combined with protein interaction databases (String, GeneMANIA) identified nine E3 ubiquitin ligases, and molecular docking predicted UBR4, HUWE1, TRIM21, and RAD18 as potential candidates (Fig. 6C). Co-IP experiments confirmed the interaction between TRIM21 and HSPD1 in A549^EPR^ cells (Fig. 6D). TRIM21 overexpression in A549^EPR^ cells reduced HSPD1 protein levels (Fig. 6E). and accelerated degradation of HSPD1 post-CHX treatment (Fig. 6F).

We next identified the TRIM21-regulated ubiquitination sites of HSPD1. MS analysis suggested that K87, K157, K160, and K405 could serve as potential ubiquitination sites for HSPD1. HSPD1 plasmids harboring specific point mutations (K87R, K157R, K160R, and K405R) were constructed. Upon TRIM21 overexpression, only the ubiquitination of HSPD1 K405R remained unaltered (Fig. 6G). Further analysis showed that the K405R mutant exhibited a markedly slower degradation rate than wild-type HSPD1 upon CHX treatment, and TRIM21 overexpression failed to enhance its degradation (Fig. 6H). Collectively, these data suggest that K405 is the principal ubiquitination site of HSPD1 mediated by TRIM21.

Subsequent experiments demonstrated that O-GlcNAcylation of HSPD1 modulates its ubiquitination. Co-IP illustrated that the T320A mutant exhibited pronounced ubiquitination compared with wild-type HSPD1 (Fig. 6J). These data indicate that the T320A mutation facilitates HSPD1 ubiquitination, with TRIM21 exhibited a greater affinity for T320A, which culminated as increased ubiquitination (Fig. 6I, J). These results confirm that T320A enhances HSPD1 degradation by promoting TRIM21-mediated ubiquitination and the proteasomal pathway.

### O-GlcNAcylation at the T320 site enhances HSPD1 stability and activates apoptosis modulation

**Fig. 7:**
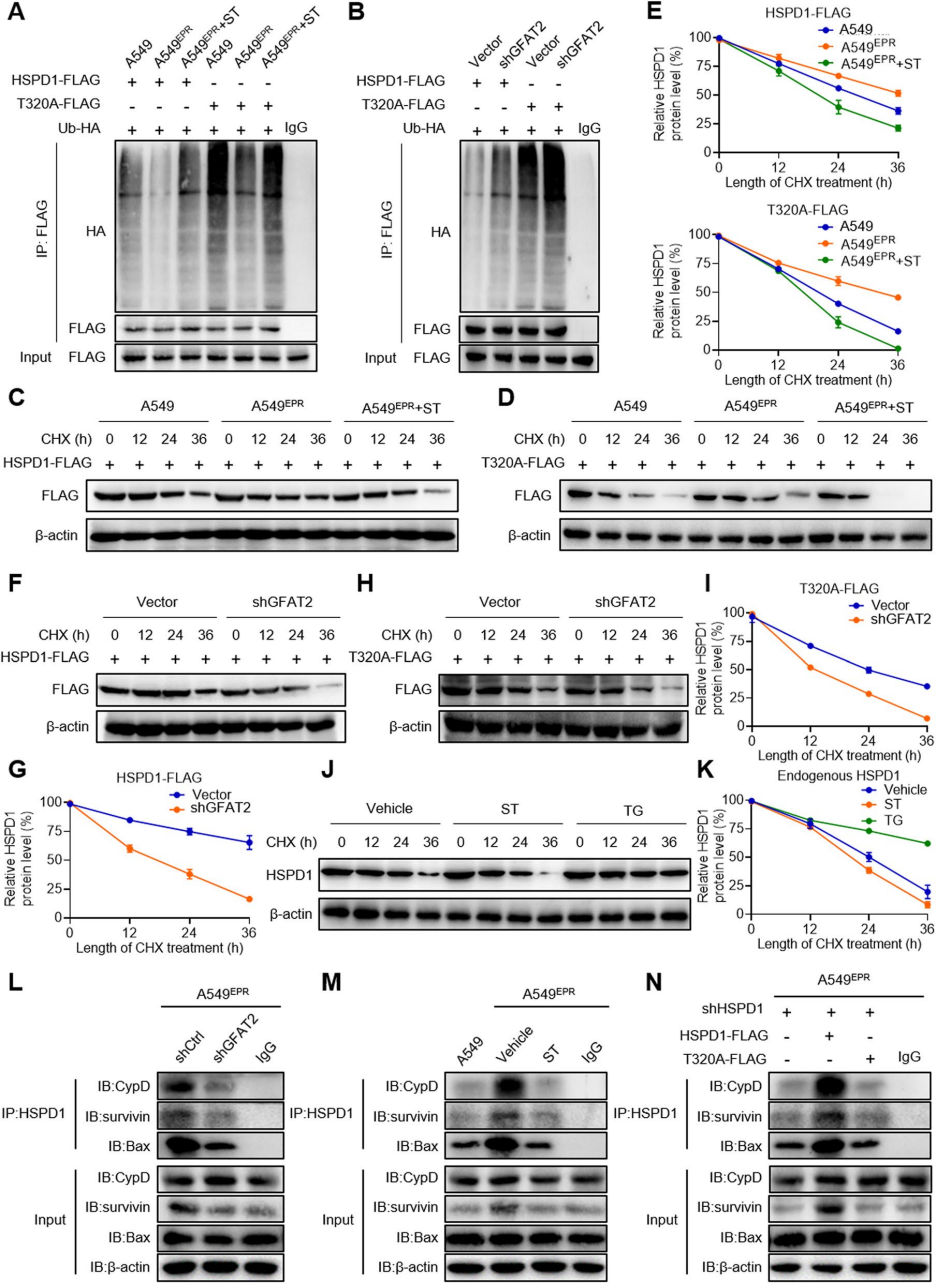
O-GlcNAcylation at the T320 site enhances HSPD1 stability and activates downstream signaling pathways. **A** HSPD1 ubiquitination in A549^EPR^ cells in the presence of HA-tagged ubiquitin (Ub-HA). **B** HSPD1 ubiquitination in *GFAT2*-knockdown cells in the presence of Ub-HA. **C**–**D** Half-life and quantitative analysis of FLAG-tagged (**C**) wild-type (WT) HSPD1 and (**D**) T320A mutant HSPD1 in A549^EPR^ cells. **E** Quantitative chart of **C**–**D**. **F**–**I** Half-life of (**F**) HSPD1-FLAG and (**H**) T320A-FLAG in *GFAT2*-knockdown cells, densitometric analyses are shown in (**G**) and **I**. **J**–**K** Half-life of endogenous (**J**) HSPD1 in A549^EPR^ cells treated with thiamet G (TG) or ST045849 (ST), **K** HSPD1 levels were analyzed using densitometric software. **L**–**N** Co-IP was performed with HSPD1 downstream target protein antibody in A549^EPR^ cells. Data shown as mean ± SEM (*n* = 3 biological replicates)

To evaluate the effects of HSPD1 O-GlcNAcylation, both parental and A549^EPR^ cells were co-transfected with ubiquitin-HA (Ub-HA) with either HSPD1-FLAG or T320A-FLAG plasmids, followed by ST treatment. Immunoprecipitation assays were performed to elucidate the ubiquitination status of HSPD1. In contrast to parental cells, A549^EPR^ cells exhibited a diminished ubiquitination of HSPD1, which remained largely unaltered by OGT inhibition via ST (Fig. 7A). Moreover, within A549^EPR^ cells, the ubiquitination of HSPD1 surged following the T320A mutation. This indicated that O-GlcNAcylation at the T320 locus may serve to mitigate ubiquitination (Fig. 7A). In addition, *GFAT2*-knockdown A549^EPR^ cells displayed increased HSPD1 ubiquitination, and the T320A mutation further amplified this effect (Fig. 7B). These observations suggest that reduced ubiquitination of HSPD1 in chemoresistant cells correlates with an elevation in O-GlcNAcylation, a process driven by GFAT2 overexpression.

To investigate whether O-GlcNAcylation-induced attenuation of ubiquitination enhances the stability of the HSPD1 protein, HSPD1-FlAG or T320A-FlAG were expressed in parental and A549^EPR^ cells, thus inhibiting protein synthesis through CHX. The half-life of HSPD1 in drug-resistant cells exceeded 24 h, whereas in parental and A549^EPR^ cells subjected to OGT inhibition, it ranged from 12 to 24 h (Fig. 7C, E). The half-life of the T320A mutant was below 24 h in drug-resistant cells (Fig. 7D, E). These findings imply that heightened T320 O-GlcNAcylation in chemoresistant NSCLC cells stabilizes HSPD1 protein.

*GFAT2* knockdown in A549^EPR^ cells led to a reduction in O-GlcNAcylation at the T320 site of the HSPD1 protein, shortening the half-life of wild-type HSPD1 from more than 24 h (control) to 12–24 h. The T320A mutant’s half-life further decreased to less than 12 h after knockdown (Fig. 7F–I). OGA inhibition (TG treatment) prolonged the half-life of endogenous HSPD1 to 36 h, whereas OGT inhibition (ST treatment) shortened it to 12 h (Fig. 7J, K). Collectively, O-GlcNAcylation at T320 of HSPD1 enhances its protein stability, with opposite effects observed under reduced modification.

The effects of GFAT2 on the intricate signalling pathways governed by HSPD1 were investigated. GFAT2 ablation markedly diminished the association of HSPD1 with cyclophilin D (CypD), survivin, and Bax when compared with that in the control cohort (Fig. 7L). In chemoresistant cells, the interaction between HSPD1 and CypD, survivin, and Bax were substantially augmented relative to parental cells, and this was effectively curtailed by ST (Fig. 7M). To further elucidate the influence of O-GlcNAcylation on the downstream Depletion of HSPD1 in A549^EPR^ cells followed by re-expression of HSPD1 or T320A revealed that HSPD1 re-expression restored the interaction with CypD, survivin, and Bax, whereas T320A yielded negligible effects (Fig. 7N). In summary, HSPD1 O-GlcNAcylation in NSCLC chemoresistant cells activates downstream anti-apoptotic pathways.

### O-GlcNAcylation at the T320 site of HSPD1 mediates chemotherapy resistance in NSCLC

**Fig. 8:**
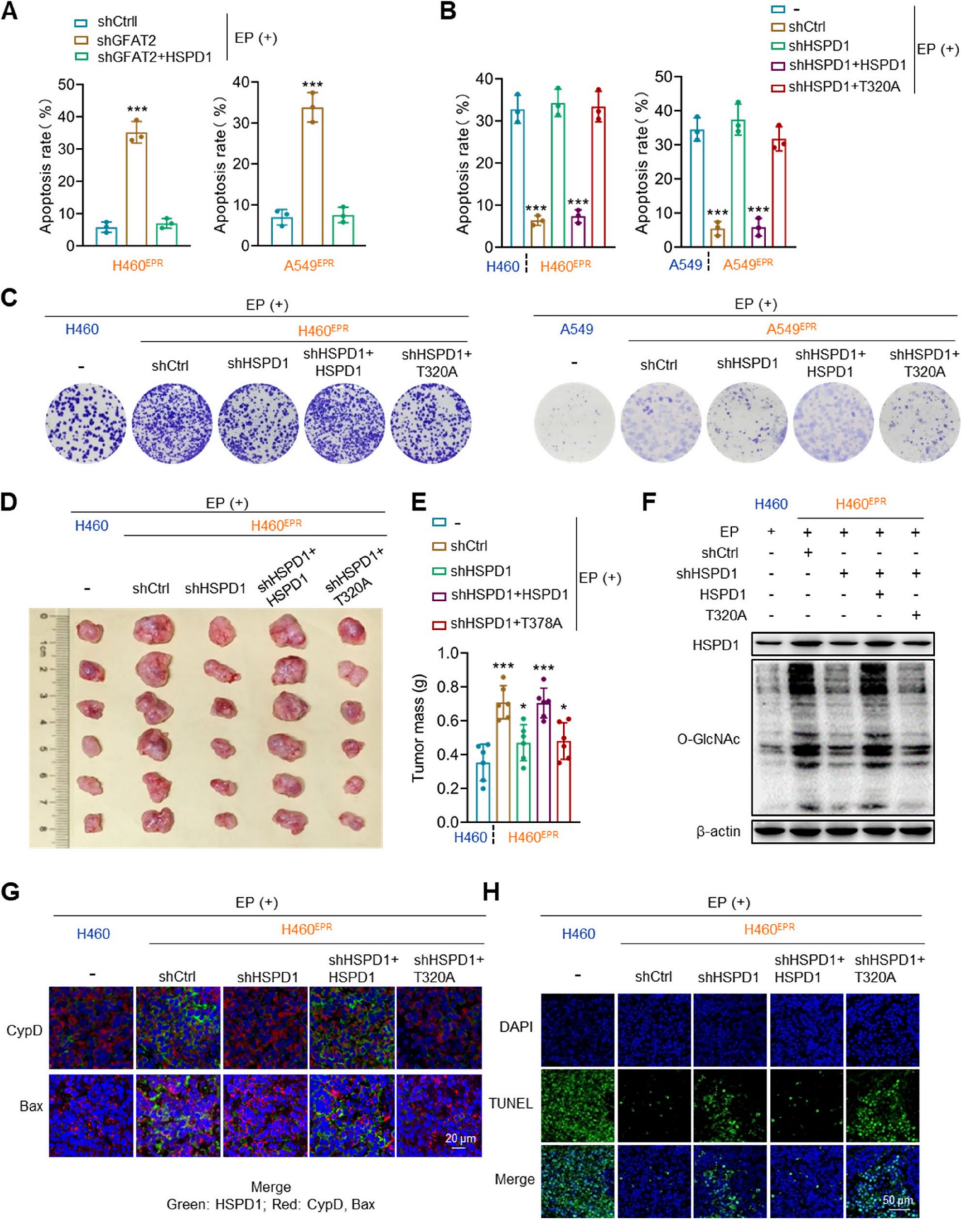
O-GlcNAcylation at the T320 Site of HSPD1 mediates chemotherapy resistance in NSCLC cells. **A** Apoptosis assay of EP-resistant cells transfected with shCtrl, shGFAT2, or shGFAT2 + HSPD1, followed by EP treatment. **B**, **C** Apoptosis assay (**B**) and colony formation assay (**C**) of parental and EP-resistant cells transduced with shCtrl, shHSPD1, shHSPD1 + HSPD1, or shHSPD1 + T320A, followed by EP treatment. **D**–**E** Tumor formation in nude mice inoculated with H460 or H460^EPR^ cells transduced with shCtrl, shHSPD1, shHSPD1 + HSPD1, or shHSPD1 + T320A, followed by EP treatment. **F** Immunoblotting analysis of O-GlcNAcylation and HSPD1 in tumors from **D**. **G** Subcellular co-localization of HSPD1 and CypD, or Bax in tumors from (**D**) was determined using immunofluorescence staining. **H** TUNEL staining of tumors from **D**. Data shown as mean ± SEM (*n* = 3 biological replicates for cell experiments; *n* = 6 biological replicates for animal experiments). *p < 0.05, ***p < 0.001

We further validated HSPD1 as the key functional mediator via functional rescue experiments in EP-resistant cells. Specifically, knocking down GFAT2 in H460^EPR^ and A549^EPR^ cells restored their sensitivity to EP, confirming the role of GFAT2 in driving resistance. Critically, HSPD1 overexpression abrogated this sensitizing effect, demonstrating that HSPD1 acts downstream of GFAT2 to directly mediate EP resistance (Fig. 8A, S7A). To explore the effects of GFAT2-mediated HSPD1 O-GlcNAcylation in EP susceptibility, HSPD1 was depleted in H460^EPR^ and A549^EPR^ cells, followed by re-expression of HSPD1 or T320A. Cellular investigations unveiled that *HSPD1* knockdown amplified the inhibitory effects of EP on colony formation and apoptosis, whereas HSPD1 re-expression countered these effects. T320A re-expression did not substantially alter the outcomes (Fig. 8B, C, S7B).

We next established a subcutaneous xenograft model to examine the effects of EP on HSPD1-depleted H460^EPR^ tumors with or without subsequent HSPD1 or T320A re-expression. HSPD1 depletion intensified the inhibitory effects of EP on the weight of drug-resistant tumors. HSPD1 re-expression nullified this effect, whereas T320A re-expression had no considerable impact (Fig. 8D, E).

Western blotting of xenograft tumors corroborated that chemoresistant tumor tissues remained elevated GFAT2 and O-GlcNAcylation levels following EP treatment. Furthermore, the depletion and restoration of HSPD1 in tumor tissues was validated via immunoblotting (Fig. 8F). Immunofluorescence assays revealed that chemoresistant tumors exhibited increased co-localization of HSPD1 with CypD and Bax, along with a diminished TUNEL positivity (Fig. 8G, H).

HSPD1 depletion curtailed this co-localization and increased TUNEL positivity, whereas HSPD1 re-expression restored the co-localization and reduced TUNEL positivity. In contrast, T320A re-expression showed no notable effect (Fig. 8G, H). These findings provide both in vivo and in vitro evidence that O-GlcNAcylation at T320 of HSPD1 drives chemotherapy resistance in NSCLC cells.

## Discussion

Previous studies on chemotherapy resistance in NSCLC have primarily focused on mechanisms underlying resistance to single drugs (etoposide, cisplatin), elucidating the complex drivers of monotherapy resistance and identifying the crucial roles of DNA repair pathways, cell cycle regulation, and apoptosis evasion. For example, Robert et al. found that higher levels of the DNA repair protein excision repair cross-complementation 1 correlates with cisplatin resistance in NSCLC [14], while increased expression of the anti-apoptotic protein Bcl-2 led to etoposide resistance [15]. However, these studies have not fully addressed the complex resistance mechanisms induced by combination therapies, which better reflects the realities of clinical treatments. Hence, this study adopts an alternative approach by exploring combined resistance to etoposide and cisplatin, thereby providing a model of chemotherapy resistance that is more aligned with clinical practice.

Through the establishment of a xenograft mouse model that faithfully replicates EP combination therapy, we uncovered a complex interplay among metabolic pathways, particularly the HBP, and chemoresistance development in NSCLC. The HBP is a branch of glucose metabolism and plays a vital role in the production of amino sugars, with approximately 3–5% of glucose funnelled into this pathway to create UDP-GlcNAc [7]. The involvement of HBP metabolic enzymes in cancer biology have been extensively explored [16, 17]. GFAT, a key HBP enzyme, acts as the first rate-limiting factor governing HBP and flux [18]. Our study demonstrated marked GFAT2 overexpression in NSCLC xenografts and EP-resistant cells. GFAT2 depletion increased EP’s inhibitory effects on resistant cell colony formation, apoptosis induction, and DNA damage, and significantly reduced the weight and volume of EP-resistant xenografts, identifying GFAT2 as a critical mediator of NSCLC chemoresistance.

Increasing understanding of NSCLC chemoresistance has highlighted the importance of metabolic adaptations that enable tumor survive under therapeutic pressure [19]. Most prior studies have focused on elucidating genetic mutations and signalling pathways underlying resistance, whereas metabolic reprogramming has emerged as a pivotal element [20]. Here, we identified a profound alteration in metabolic pathways, notably the upregulation of proteins associated with alanine, aspartate, and glutamate metabolism, which are essential for the HBP [21, 22]. This metabolic shift underscores the critical role of the HBP in energy metabolism and the biosynthetic processes supporting NSCLC cell proliferation and survival during chemotherapy. Our data revealed a compelling correlation between aberrant expression of GFAT2 and increased O-GlcNAcylation in chemoresistant NSCLC cells. This post-translational modification stabilizes HSPD1, thereby activating the anti-apoptotic pathways that contribute to chemotherapy resistance. These findings enrich the metabolic understanding of NSCLC chemoresistance, position GFAT2 as a promising target for therapeutic intervention, and lay the groundwork for innovative strategies to overcome EP resistance, with the potential to enhance treatment efficacy and patient outcomes.

Elevated HBP flux is tightly linked to tumor metabolic reprogramming [23]. The expression levels of HBP metabolic enzymes are notably elevated in tumors, which drives increased synthesis of UDP-GlcNAc [24]. As the terminal HBP product and key donor substrate for O-GlcNAcylation, UDP-GlcNAc concentration is positively associated with O-GlcNAcylation levels. This process, which involves the addition of a single GlcNAc moiety to serine/threonine residues on proteins, regulates cell metabolism, gene expression, and signal transduction, ultimately influencing protein function and facilitating cancer initiation and progression [25], making the HBP and O-GlcNAcylation attractive anti-cancer targets. Metabolomic analysis in our study revealed profound metabolic reprogramming in EP-resistant NSCLC cells, with upregulation of the HBP, purines and pyrimidines synthesis pathways, and increased levels of O-GlcNAcylation-associated metabolites (GlcN-6-P, GlcNAc-1-P, UDP-GlcNAc). EP-resistant cells also exhibited markedly increased O-GlcNAcylation, which was reduced by GFAT2 depletion. O-GlcNAcylation is regulated by OGT (GlcNAc addition) and OGA (GlcNAc removal), and OGT-mediated modification is closely tied to intracellular UDP-GlcNAc levels—a nutrient sensor that integrating metabolic and signalling pathways. Using OGT/OGA inhibitors, we confirmed that GFAT2-driven O-GlcNAcylation relies on the post-translational regulation of OGT and OGA proteins. GFAT2 enhanced OGT expression while suppressing OGA expression, thereby influencing intracellular protein O-GlcNAcylation and promoting EP resistance in NSCLC.

O-GlcNAcylation is crucial for regulating apoptosis and DNA damage [26–28]. For instance, receptor-interacting serine/threonine-protein kinase 1, a key regulator of the balance between cell death and survival, is poorly expressed during the development of sunitinib resistance [29]. To investigate the mechanism by which O-GlcNAcylation affects chemosensitivity in NSCLC, we performed MS analysis of the OGT interactome and validated HSPD1 as a specific substrate of OGT via exogenous and endogenous Co-IP assays. HSPD1 is a mitochondrial chaperone protein encoded by nuclear DNA that is essential for maintaining mitochondrial protein balance and substantially influences apoptosis regulation [13]. It localizes to mitochondria and directly binds CypD, a component of the mitochondrial permeability transition pore [30], to prevent abnormal opening of pores and tumor cell apoptosis.

HSPD1 also interacts with cell cycle and apoptosis regulator 2, p53, and survivin to exert pro-survival capabilities [31, 32], with mitochondrial survivin inhibiting procaspase activation and HSPD1 binding Bax to inhibits its pro-apoptotic activity, thus promoting cell survival and tumor progression [31]. Various post-translational modifications, such as ubiquitination and acetylation, are essential for the proper functioning of HSPD1 [33]. In this study, OGT specifically interacted with the apical domain of HSPD1 to enhance its O-GlcNAcylation. We predicted potential sites and identified threonine 54 and threonine 320 as candidates residues, with Co-IP confirming Thr320 as the primary O-GlcNAcylation site. To our knowledge, this is the first report documenting the O-GlcNAcylation of HSPD1, thus highlighting its potential as a therapeutic target in NSCLC.

The specific function of HSPD1 following O-GlcNAcylation remains poorly characterized. HSPD1 undergoes ubiquitination in necrotic monocytes [34], yet the crosstalk between its O-GlcNAcylation and ubiquitination is not well defined. Our findings suggest that the E3 ubiquitin ligase TRIM21 causes ubiquitination of the K405 site of HSPD1, leading to its degradation via the proteasome pathway. OGT-mediated O-GlcNAcylation of HSPD1 decreases TRIM21-mediated degradation of HSPD1. Notably, the T320A mutation, which abolishes O-GlcNAcylation, results in increased ubiquitination of HSPD1, thereby enhancing its degradation. Furthermore, O-GlcNAcylation of HSPD1 at Thr320 facilitates its binding to CypD, survivin, and Bax, thereby activating the downstream anti-apoptotic signalling pathways associated with HSPD1. Additionally, the in vivo and in vitro experiments in this study demonstrate that O-GlcNAcylation at Thr320 of HSPD1 plays a crucial role in mediating the anti-apoptotic effects observed in resistant cells, thereby providing insights into the development of EP resistance in NSCLC.

While our study defines a definitive GFAT2-HSPD1 T320 O-GlcNAcylation axis driving EP resistance in NSCLC, it lacks direct clinical evidence linking GFAT2 expression or HSPD1 T320 O-GlcNAcylation to chemotherapy response in patient samples. And the specific molecular mechanisms by which GFAT2 regulates OGT and OGA remain unelucidated. Validation of this axis in patient-derived xenograft models and analysis of well-annotated clinical samples will establish its clinical relevance, clarify the correlation between GFAT2/HSPD1 T320 O-GlcNAcylation and EP response, and provide robust clinical evidence for GFAT2/HSPD1 T320 O-GlcNAcylation as a prognostic biomarker for NSCLC chemoresistance.

## Conclusions

**Fig. 9:**
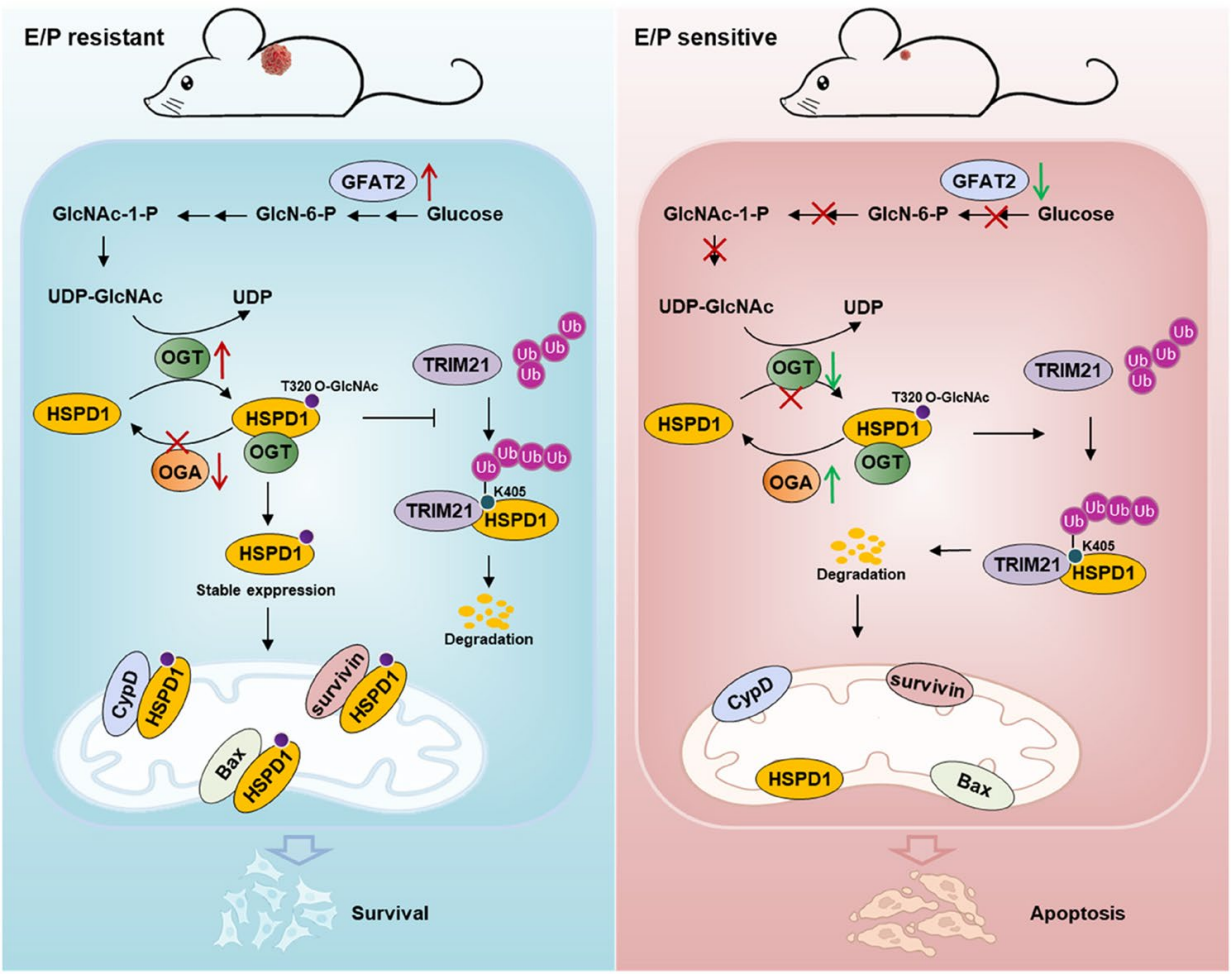
Schematic diagram of the mechanism by which GFAT2 increases HSPD1 O-GlcNAcylation to promote chemotherapy resistance in NSCLC

In conclusion, this study established a chemotherapy-resistant xenograft mouse model mimicking clinical NSCLC regimens and employed proteomic and metabolomic analyses‌ to investigate the molecular mechanisms underlying chemoresistance (Fig. 9). The study identified the overexpression of GFAT2, a key enzyme in the HBP, in resistant NSCLC models, thereby revealing its role in driving UDP-GlcNAc overproduction and subsequent O-GlcNAcylation of HSPD1‌. This post-translational modification of HSPD1 is functionally linked to enhanced resistance to chemotherapy. Moreover, the study revealed a previously unknown mechanism in which GFAT2 increased UDP-GlcNAc production and O-GlcNAcylation and delineated the functional importance of HSPD1 O-GlcNAcylation in facilitating chemotherapy resistance in NSCLC. The results of this study are expected to offer new therapeutic strategies to combat chemotherapy resistance in NSCLC, ultimately enhancing patient treatment outcomes.

## Supplementary Information

Supplementary Material 1.

## Data Availability

No datasets were generated or analysed during the current study.

## Abbreviations

NSCLC: Non-small cell lung cancer
EP: Etoposide/cisplatin
GFAT2: Glutamine-fructose-6-phosphate transaminase 2
HBP: Hexosamine biosynthetic pathway
UDP-GlcNAc: Uridine diphosphate-GlcNAc
HSPD1: Heat shock protein family D member 1
TRIM21: Tripartite motif containing 21
CDX: Cell line-derived xenograft
γH2AX: Gamma H2A histone family member X
OGT: O-GlcNAc transferase
OGA: O-linked N-acetylglucosaminase
GlcN-6-P: Glucosamine-6-phosphate
GlcNAc-1-P: N-acetylglucosamine-1-phosphate
ST: OGT inhibitor ST045849
TG: OGA inhibitor thiamet G
IP/MS: Immunoprecipitation coupled with mass spectrometry
Co-IP: Co-immunoprecipitation
CHX: Cycloheximide
CQ: Chloroquine
