# Supplementary material for: GFAT2-mediated HSPD1 O-GlcNAcylation drives chemotherapy resistance in non-small cell lung cancer
Source: J Exp Clin Cancer Res. 2026 Feb 24;45:110. doi: 10.1186/s13046-026-03674-x (PMC13141267; doi:10.1186/s13046-026-03674-x)
Supplement: Supplementary file 1 — Supplementary Material 1. [file 13046_2026_3674_MOESM1_ESM.docx]

**Supplementary Materials for**

**GFAT2-mediated HSPD1 O-GlcNAcylation drives chemotherapy resistance in non-small cell lung cancer**

Man Zhu^1,2^, Xiaoyu Tang^2,3^, Zeren Zhu^2,3^, Wenjun Tang^2,3^, Yumeng Cheng^2,3^, Wenjuan Tang^2,3^, Qianqian Zhang^2,3^, Longyu Qin^2,3^, Yu Yao^1^, Yanmin Zhang^1,2, *^

^1^Department of Medical Oncology, the First Affiliated Hospital of Xi’an Jiaotong University, Xi’an 710061, P. R. China

^2^School of Pharmacy, Health Science Center, Xi’an Jiaotong University, Xi’an 710061, P.R. China

^3^State Key Laboratory of Shaanxi for Natural Medicines Research and Engineering, Xi'an 710061, P.R. China

**Supplementary materials**

This PDF file includes:

Figure. S1 to S7

Materials and Methods

**Supplementary figures and figure legends**

**
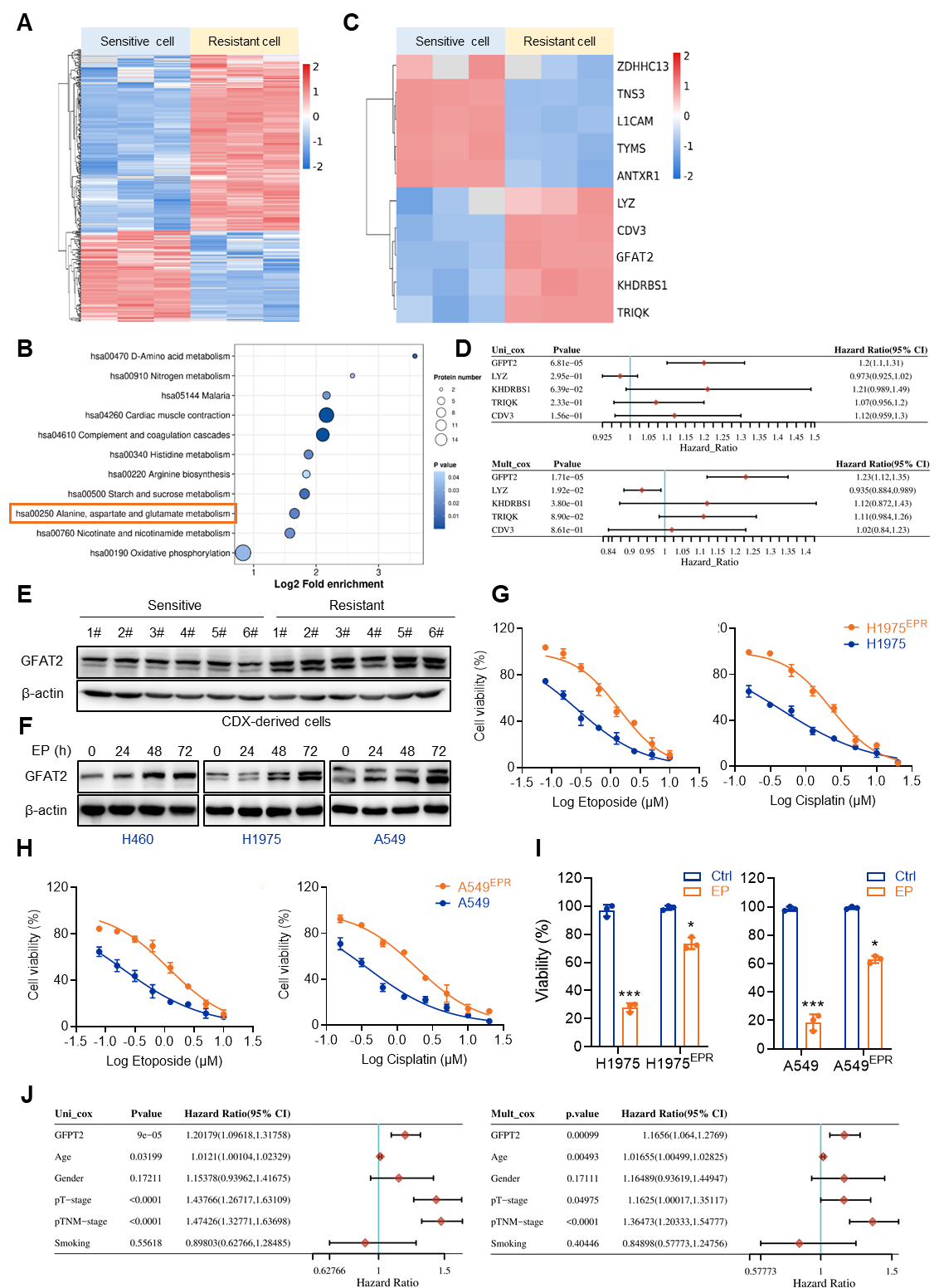
**

**Figure S1.** (A) Heatmap of differential proteins between chemotherapy resistant cells and sensitive cells. (B) KEGG pathway bubble plot of differential proteins between chemoresistant cells and chemosensitive cells. (C) Heatmap of the top 5 proteins arranged by differential multiples. Red represents high expression, blue represents low expression, and gray represents unquantifiable samples. (D) Forest plots displaying hazard ratios with 95% confidence intervals and corresponding *P*-values for key genes from univariate and multivariate Cox proportional hazards regression analyses. (E) Immunoblotting analysis of GFAT2 in chemotherapy sensitive and resistant tumors. (F) Immunoblotting analysis of GFAT2 in NSCLC parental cell lines treated with EP. (G) Relative cell viability of H1975 and H1975^EPR^ cells treated upon different concentrations of etoposide and cisplatin for 48 h. (H) Relative cell viability of A549 and A549^EPR^ cells treated upon different concentrations of etoposide and cisplatin for 48 h. (I) Relative viability of H1975 and H1975^EPR^ cells (0.16 μM etoposide and 0.24 μM cisplatin), A549 and A549^EPR^ cells (0.12 μM etoposide and 0.18 μM cisplatin) following 48 h of EP treatment in vitro. (J) Identiﬁcation of independent risk factors for predicting overall survival in NSCLC. Data shown as mean ± SEM (n=3 biological replicates for cell experiments). **p* < 0.05, ****p* < 0.001.


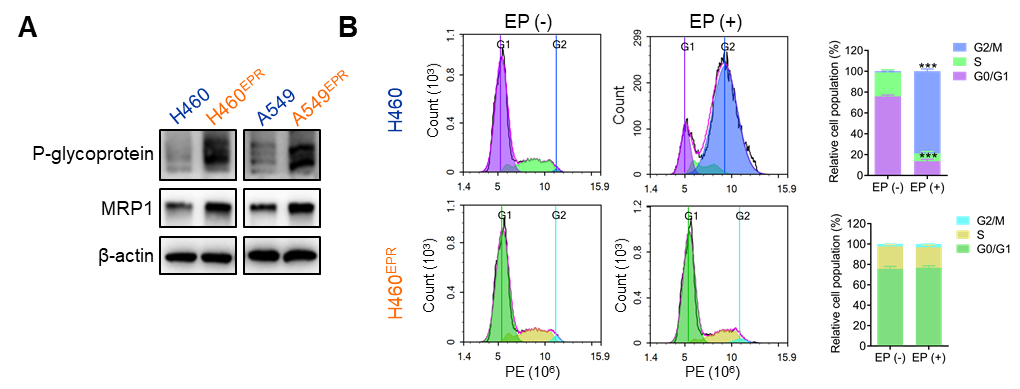


**Figure S2.** (A) Western blotting showing the protein expression levels of P-glycoprotein and MRP1 in parental and EP-resistant NSCLC cell lines. (B) Flow cytometry analysis of cell cycle distribution in parental and EP-resistant cells with or without EP treatment. Data shown as mean ± SEM (n=3 biological replicates for cell experiments). ****p* < 0.001.


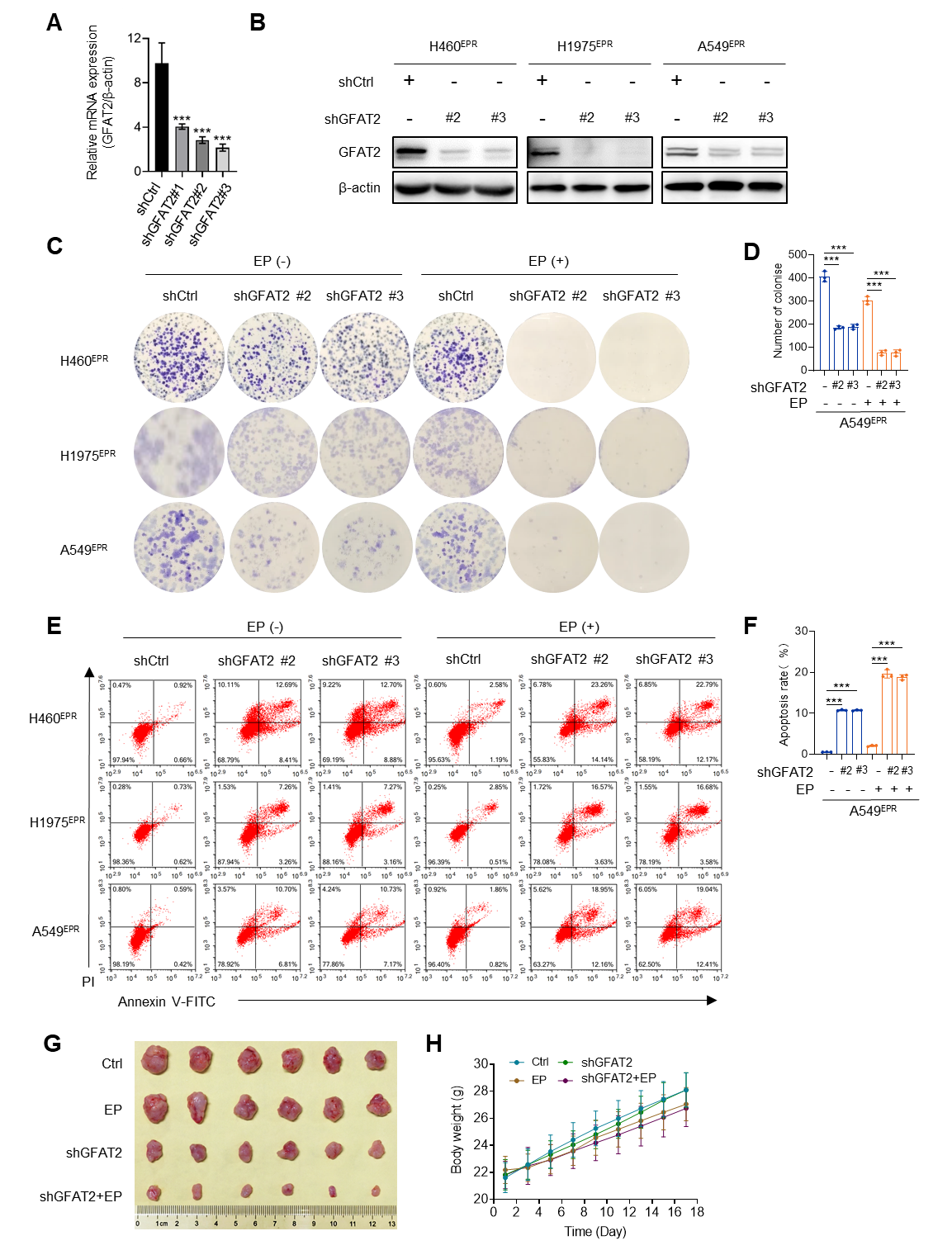


**Figure S2.** (A) GFAT2 mRNA expression in H460^EPR^ cells after GFAT2 knockdown. (B) Immunoblotting analysis of GFAT2 in H460^EPR^, H1975^EPR^ and A549^EPR^ cells with GFAT2 knockdown. (C-D) Colony formation capacity of A549^EPR^ cells with GFAT2 knockdown, with or without EP treatment. (E-F) Apoptosis rate of A549^EPR^ cells with GFAT2 knockdown in the presence or absence of EP. (G-H) Eﬀect of GFAT2 knockdown on tumor growth in H460^EPR^ cells with or without EP treatment. Mice were treated with vehicle control or EP (10 mg/kg). Data shown as mean ± SEM (n=6 biological replicates for RT-qPCR experiments; n=6 biological replicates for animal experiments). ****p* < 0.001.


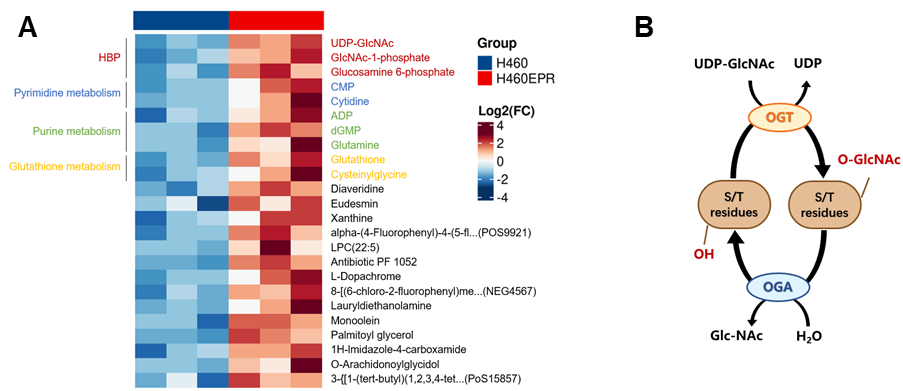


**Figure S4.** (A) Heatmap of metabolites between chemoresistant cells and chemosensitive cells. (B) Working model whereby O-GlcNAcylation cycle.


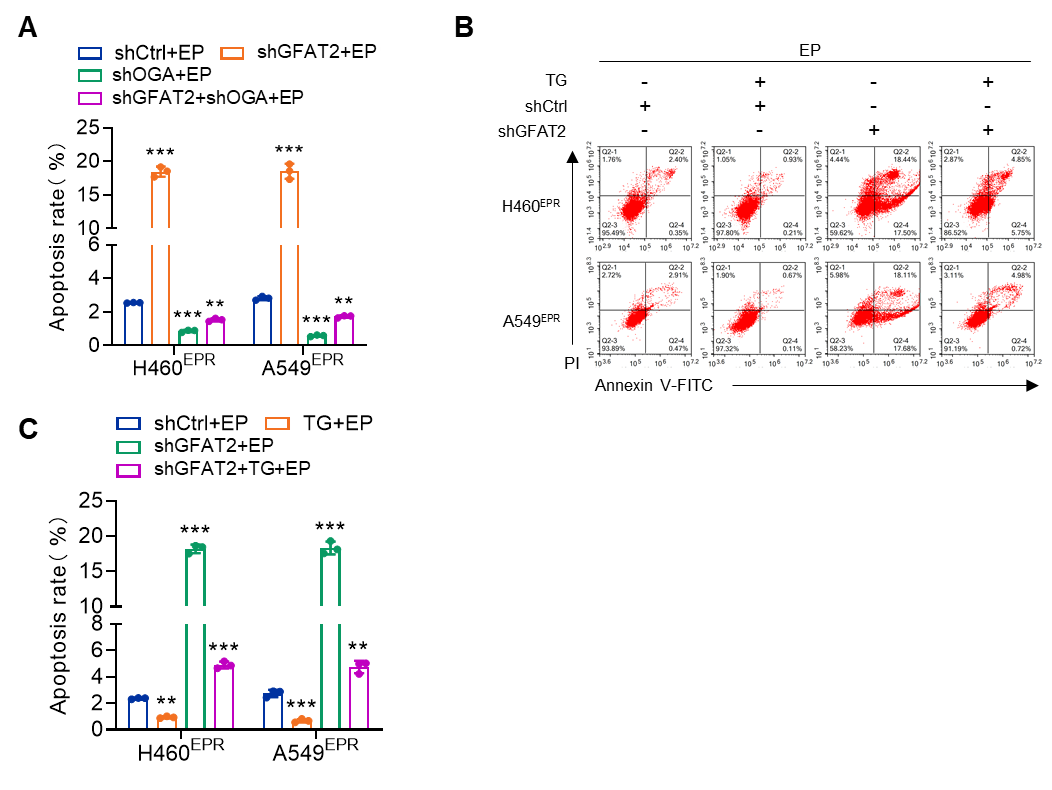


**Figure S5.** (A) Apoptosis rate of H460^EPR^ and A549^EPR^ cells with OGA and GFAT2 knockdown in the presence of EP. (B-C) Apoptosis rate of H460^EPR^ and A549^EPR^ cells with TG or GFAT2 knockdown in the presence of EP. Data shown as mean ± SEM (n = 3 biological replicates). ***p* < 0.01, ****p* < 0.001.

**
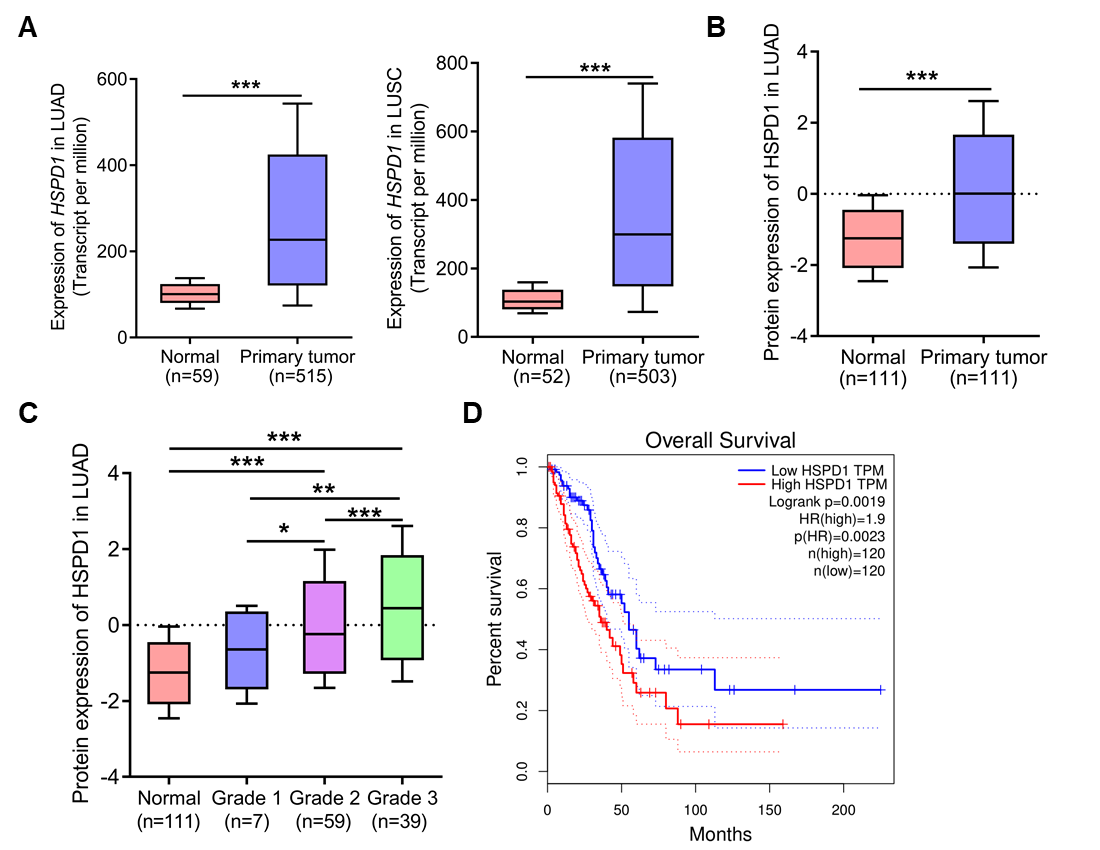
**

**Figure S6.** (A) Box plots showing HSPD1 transcript levels in normal lung tissue and primary tumor tissue from patients with lung adenocarcinoma (LUAD; n = 59 normal, n = 515 primary tumor) and lung squamous cell carcinoma (LUSC; n = 52 normal, n = 503 primary tumor). (B) Box plot comparing HSPD1 protein expression between normal lung tissue (n = 111) and primary LUAD tumors (n = 111). (C) Box plot demonstrating HSPD1 protein expression levels across increasing tumor grades (Grade 1–3) in LUAD patients, relative to normal lung tissue. (D) Kaplan-Meier overall survival curve for LUAD patients stratified by HSPD1 transcript levels. The log-rank test revealed significantly poorer survival in the high HSPD1 expression group. **p* < 0.05, ***p* < 0.01, ****p* < 0.001.


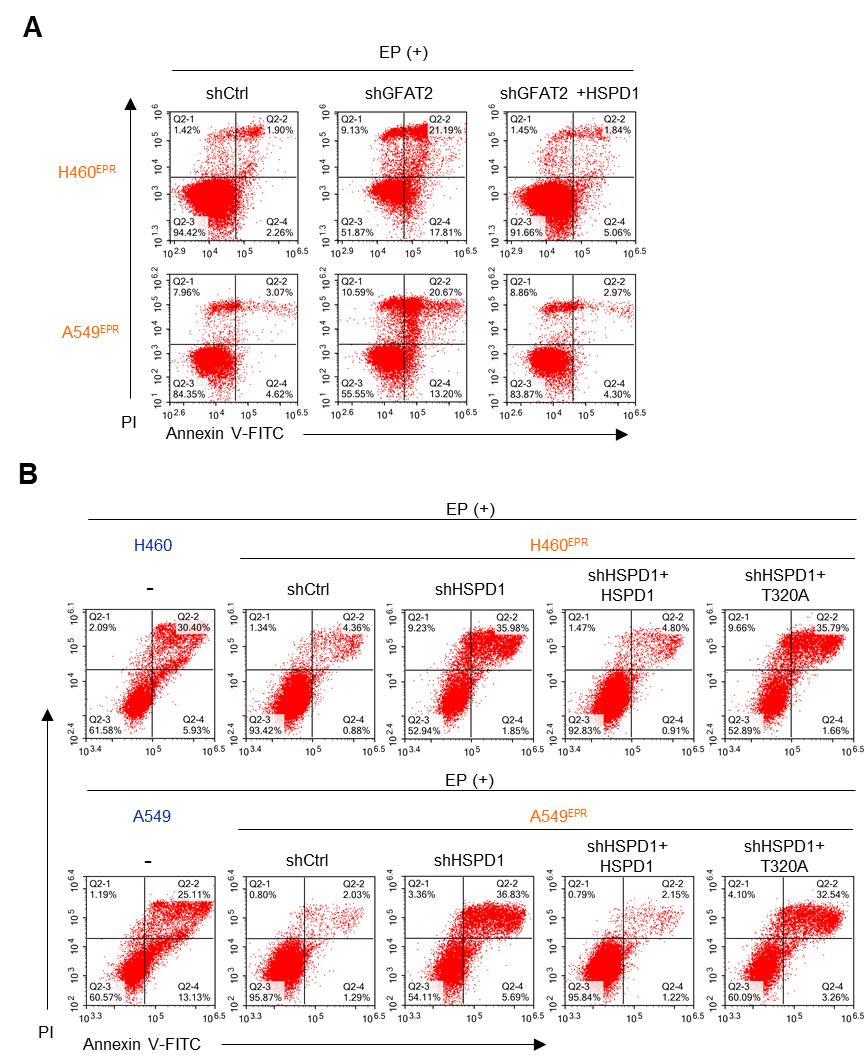


**Figure S7.** (A) Apoptosis assay of EP-resistant cells transfected with shCtrl, shGFAT2, or shGFAT2+HSPD1, followed by EP treatment. (B) Apoptosis assay of parental and EP-resistant cells transduced with shCtrl, shHSPD1, shHSPD1+HSPD1, or shHSPD1+T320A, followed by EP treatment.

**Supplementary methods**

**Lentiviral transduction**

Negative control virus and *GFAT2* knockdown lentivirus were transfected using Hitrans G virus co-infection reagent according to the manufacturer’s instructions. After 16 h of transfection, the virus was removed and replenished with fresh complete medium. After 48 h of transfection, the expression of enhanced green fluorescent protein was observed under a microscope. When the positive rate of green fluorescence was high, puromycin with a final concentration of 1 μg/mL was added for continuous subculture, and the H460^EPR^ cell model with stable *GFAT2* knockdown was constructed for subsequent animal experiments.

**Cell viability assay**

Cells in the logarithmic growth phase were collected and seeded into 96-well plates at a density of 2 × 10^4^ cells/well. Various treatments were performed after incubation for 24 h. After treatment, cells were cultured with serum-free medium and 3-(4,5-dimethylthiazol-2-yl)-2,5-diphenyltetrazolium bromide for 4 h, and 150 µL of dimethyl sulfoxide was then added for 15 min. The absorbance was measured at 490 nm using a microplate reader (Bio-Rad, Hercules, CA, USA).

**Immunoprecipitation**

The cells were seeded into 100 mm culture dishes at a density of 6 × 10^5^ cells per dish, and cultured overnight for 24 h before various treatments. According to the instructions of the Immunoprecipitation kit KIP-2, the control and treated histone lysates were obtained. After BCA quantification, equal amounts of protein were taken for co-immunoprecipitation. The protein lysates were incubated with the indicator antibody or IgG control primary antibody respectively (4°C, 50 rpm/min, 12 h), the immunoprecipitated complexes were eluted, and the indicator antibody was used for immunoblotting. The antibodies used in this experiment included PPIF (18466-1-AP, Proteintech, 1:2000), survivin (F0264, Selleck, 1:1000), and Bax (GB154122, Servicebio, 1:1000).

**Immunofluorescence staining**

Frozen sections with a thickness of 5 μm were processed as previously described. After fixation, slides were incubated with mouse anti-HSPD1 monoclonal antibody (15282-1-AP, Proteintech, 1:500) for 1 h at room temperature, and then DyLight 488 labeled goat anti-mouse antibody (diluted 1:1000, Abbkine). Then rabbit anti-PPIF (18466-1-AP, Proteintech, 1:100), survivin (F0264, Selleck, 1:500), and Bax (GB154122, Servicebio, 1:500) monoclonal antibodies were added, and goat anti-rabbit IgG-DyLight 594 (diluted 1:1000, Abbkine). Nuclei were identified by counterstaining with 4,6-diamino-2-phenylindolyl dihydrochloride (DAPI; 1 μg/mL). All slides were scanned using a scanner (Pannoramic MIDI, 3DHISTECH, Hungary). Using C.V.2.4 software captures the digital image of each fluorescence channel and superimposes it to show the specific antibody staining. The fluorescent green signal represents the expression of HSPD1, the red signal represents the expression of PPIF, survivin, and Bax, and the merged yellow orange signal shows the co-localization on single cells.
